# Reprogramming stem cells in regenerative medicine

**DOI:** 10.1002/SMMD.20220005

**Published:** 2022-12-25

**Authors:** Jiayi Mao, Qimanguli Saiding, Shutong Qian, Zhimo Liu, Binfan Zhao, Qiuyu Zhao, Bolun Lu, Xiyuan Mao, Liucheng Zhang, Yuguang Zhang, Xiaoming Sun, Wenguo Cui

**Affiliations:** ^1^ Department of Plastic and Reconstructive Surgery Shanghai Ninth People's Hospital Shanghai Jiao Tong University School of Medicine Shanghai China; ^2^ Department of Orthopaedics Shanghai Key Laboratory for Prevention and Treatment of Bone and Joint Diseases Shanghai Institute of Traumatology and Orthopaedics Ruijin Hospital Shanghai Jiao Tong University School of Medicine Shanghai China

**Keywords:** cell therapies, cellular reprogramming, human disease model, induced pluripotent stem cells, tissue regeneration

## Abstract

Induced pluripotent stem cells (iPSCs) that are generated from adult somatic cells are induced to express genes that make them pluripotent through reprogramming techniques. With their unlimited proliferative capacity and multifaceted differentiation potential and circumventing the ethical problems encountered in the application of embryonic stem cells (ESC), iPSCs have a broad application in the fields of cell therapy, drug screening, and disease models and may open up new possibilities for regenerative medicine to treat diseases in the future. In this review, we begin with different reprogramming cell technologies to obtain iPSCs, including biotechnological, chemical, and physical modulation techniques, and present their respective strengths, and limitations, as well as the recent progress of research. Secondly, we review recent research advances in iPSC reprogramming‐based regenerative therapies. iPSCs are now widely used to study various clinical diseases of hair follicle defects, myocardial infarction, neurological disorders, liver diseases, and spinal cord injuries. This review focuses on the translational clinical research around iPSCs as well as their potential for growth in the medical field. Finally, we summarize the overall review and look at the potential future of iPSCs in the field of cell therapy as well as tissue regeneration engineering and possible problems. We believe that the advancing iPSC research will help drive long‐awaited breakthroughs in cellular therapy.

## Graphical abstract

Induced pluripotent stem cells (iPSC) that are generated from adult somatic cells are induced to express genes that make them pluripotent through reprogramming techniques, which may open up new possibilities for regenerative medicine to treat diseases in the future. Biotechnological, chemical, and physical modulation techniques are different reprogramming cell technologies to obtain iPSC and present their respective strengths in iPSC reprogramming‐based regenerative therapies, such as hair follicle defects, myocardial infarction, neurological disorders, liver diseases, and spinal cord injuries.

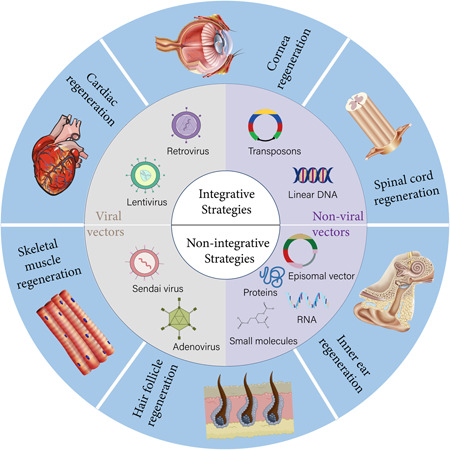

1

**Key points**

- Different reprogramming cell technologies to obtain induced pluripotent stem cells (iPSCs).
- Recent research advances in iPSCs reprogramming‐based regenerative therapies.
- Translational clinical research around iPSCs as well as their potential for growth in the medical field.
- iPSCs in the field of cell therapy as well as tissue regeneration engineering and possible problems.

## 2 INTRODUCTION

The regenerative medicine field is booming and the search for pluripotent/allopathic stem cells that can be transgerminally differentiated and safely used for therapeutic purposes is a current task of regenerative science. However, stem cells used for research or clinical applications, except for hematopoietic stem/progenitor cells (HSPCs) used for hematological applications, are mainly monoenergetic stem cells, such as mesenchymal stem cells (MSCs) derived from tissues isolated after birth, with limited differentiation potential. Because of their monoenergetic nature, they will be more widely used in the clinical setting. Cells that exhibit more pluripotency also have difficulties in clinical applications, such as the more primitive embryonic stem cells (ESCs) and induced pluripotent stem cells (iPSCs), both of which exhibit the possibility of genomic instability, teratoma formation risk, as well as immune exclusion.^1^ Hence, the only secure stem cells are monoenergetic stem cells and pluripotent stem cells that can solve the above‐mentioned problems in current regenerative medicine are urgently need.

iPSCs are multipotent stem cells derived from adult somatic cells.^2^ Discoveries of iPSCs allow the development of human ESCs‐like stem cells derived from different adult cells (e.g., fibroblasts) without destroying human embryos, thus circumventing the ethical and immune rejection problems accompanying the application of ESCs. Narrowly speaking, reprogramming means always specifically the restoration of a polypotent state to a differentiated somatic cell. It is possible to differentiate back into virtually any cell type. Research studies have shown in vivo and in vitro that cell reprogramming occurs. Natural reprogramming in vivo takes place in fertilization, giving rise to totipotent cells.^3^ Reprogramming in vitro can also be realized by various methods. In the early 20th century, Yamanaka's group first reprogrammed somatic cells by enforcing the expression of four transcription factors to iPSCs, and overexpression of cellular reprogramming factors (Oct4, Sox2, KLF4, Nanog, and c‐Myc) restored polarized cells to early stages of development, bringing breakthroughs in both biomolecular engineering and biomedicine.^4^ Following the initial pioneering work to generate iPSC via retroviral transduction of its four reprogramming genes, several other methods to obtain iPSCs were already evolved to improve the productivity and safety of the procedure. Our comprehension of PSC biology is now so advanced that it is now possible to generate most human cells in the laboratory. The generation of iPSC is being performed by various alternative methods, including nuclear transplantation, mandatory delivery of specific agents, and combinations of one or two minor molecular compounds. Nevertheless, the efficiency for all of the reprogramming methods remained as poor as 1%–5%. Thus, to enhance the reprogramming productivity of iPSC, several study teams have revised the reprogramming factors or adopted new approaches. The different reprogramming methods that are currently applied are summarized in our review (Table 1).

**TABLE 1:** Methods to reprogram stem cells

Conveyor systems			Advantages	Disadvantages	Research progress	Literature
Integrated systems	Viral systems	Retroviruses	High infection efficiency	Genome integration Low reprogramming efficiency Mutagenesis risk Infects only dividing cells	Retroviruses were first used as vectors for reprogramming factors. iPS induction was first performed with MMLV‐derived retroviral vectors, which deliver transgenes of up to 8 kb, but only into dividing cells.	Samoilova et al. (2018)^11^
		Lentiviruses	High infection efficiency Infects both dividing and non‐dividing cells	Genome integration Low reprogramming efficiency Mutagenesis risk	Lentiviral vectors demonstrate more efficient delivery of gene constructs to slowly dividing cells, such as neurons.	Wang et al. (2021)^15^
					Reprogrammed EC can be used in implantable vascular networks, and these reprogrammed cells exhibit high levels of expression of well‐defined endothelial cell markers that produce nitric oxide, critical to the vascular function in adult endothelial cells.	Morita et al. (2015)^14^
	Nonviral systems	Transposon vector	Low cost Can carry 8–10 kb transgenes	Genome integration Low reprogramming efficiency Removal requires the use of transposase	The piggyBac transposon system generates iHeps by integrating a transgene consisting of Hnf4a and Foxa3 and successfully obtains functional iHeps.	Katayama et al. (2017)^17^
					A PiggyBac transposon vector was constructed to co‐express the PB_OMKS on BFSF to establish the BFSF_OMKS cell line and reprogram the somatic cells.	Luo et al. (2022)^18^
					Identification of novel SB transposon transposase mutants and establishment of compensatory amino acid substitutions that can completely rescue the integration defects of these mutants, demonstrating that shearing and pasting of DNA transpositions can be converted into a unidirectional process by a single amino acid change.	Kesselring et al. (2020)^19^
Nonintegrated systems	Viral systems	Adenovirus	No genomic integration Avoid insertion of oncogenic mutations	Low reprogramming efficiency Multiple rounds of transfection are required Need to remove viral genome	Provides the first evidence that adenoviral OSKM delivery can induce partial reprogramming of postnatal cardiomyocytes, aiming to induce transient reprogramming of mammalian cardiomyocytes in vitro using a non‐integrating vector encoding OSKM.	Kisby et al. (2021)^20^
		Sendai virus vector	No genome integration Efficient reprogramming Deliver up to four exogenous in a single vector Avoid insertion of oncogenic mutations	Need to remove viral genome	Transformation of human pluripotent stem cells into SkM cells using a temperature‐sensitive SeV‐Myod1.	Tan et al. (2021)^22^
	Nonviral systems	Minicircle DNA	No genome integration High transfection efficiency Long ectopic expression	No self‐replication	hiPSC was constructed by transfecting simple nonviral minicircle DNA in hASCs. Minicircle DNA vectors do not contain viral DNA and thus can be repeatedly transfected with hASCs highly expressed in mammalian cells within approximately 4 weeks.	Narsinh et al. (2011)^23^
					Induction of reprogramming in mouse melanoma B16F10 cells using nonviral minicircle DNA containing four reprogramming factors OSLN, and GFP reporter gene.	Câmara et al. (2017)^24^
		Episomal vectors	No genome integration Rapid elimination of transgenes from cells	Low reprogramming efficiency Low transfection efficiency	Enrichment of transfected cells was facilitated by modifying the original vector to express spectrally separable fluorescent proteins.	Schmitt et al. (2017)^25^
					By inserting CD genes from yeast into episomal vectors, episomal vectors that eliminate free DNA in only 7 days are established and used to reprogram human fibroblasts into iPSCs. 5‐FC treatment allows for rapid and easy isolation of exogenous‐free reprogrammed cells that can be used for disease modeling and clinical applications.	Lee et al. (2019)^26^
		RNA	No genome integration Efficient reprogramming	Multiple rounds of transfection are required	Linc‐ROR promotes chondrogenic differentiation and chondrogenesis in BMSCs by acting as a competitive endogenous RNA for miR‐138 and miR‐145 and activating SOX9 expression.	Feng et al. (2021)^27^
					miR‐200c‐141 promotes reprogramming in sheep somatic cells, which is achieved by decreasing the expression of ZEB1 3′UTR and increasing the expression of E‐cadherin.	Zhang et al. (2021)^28^
		Protein	No genome integration	Low reprogramming efficiency Requires multiple rounds of transfection Time‐consuming	A new technology for using TiO_2_ nanotubes to deliver reprogrammed proteins to differentiated fibroblasts without cytotoxic effects and without affecting cell proliferation is described. Following 3 weeks of processing with protein‐bound nanotubes, somatic cells adopted an ECM‐like morphology and expressed activated Oct4‐green fluorescent protein, a pluripotency biomarker.	Cho et al. (2013)^30^
					To establish a novel reprogramming technique for reprogramming dermal fibroblasts into pluripotent cells using a single protein, FMOD, and to demonstrate that maintaining cellular localization and retention in the receptor tissue environment is equally important for establishing critical‐sized muscle tissue.	Yang et al. (2020)^31^
		Liposomal magnetofection	No genome integration Rapid reprogramming	Inefficient reprogramming	Effective conditions for generating virus‐free iPSC from MEF using different concentrations of CombiMag‐DNA nanoparticle liposomes and one or two cycles of the LMF procedure. Reprogramming time <8 days.	Park et al. (2012)^32^

**FIGURE 1:**
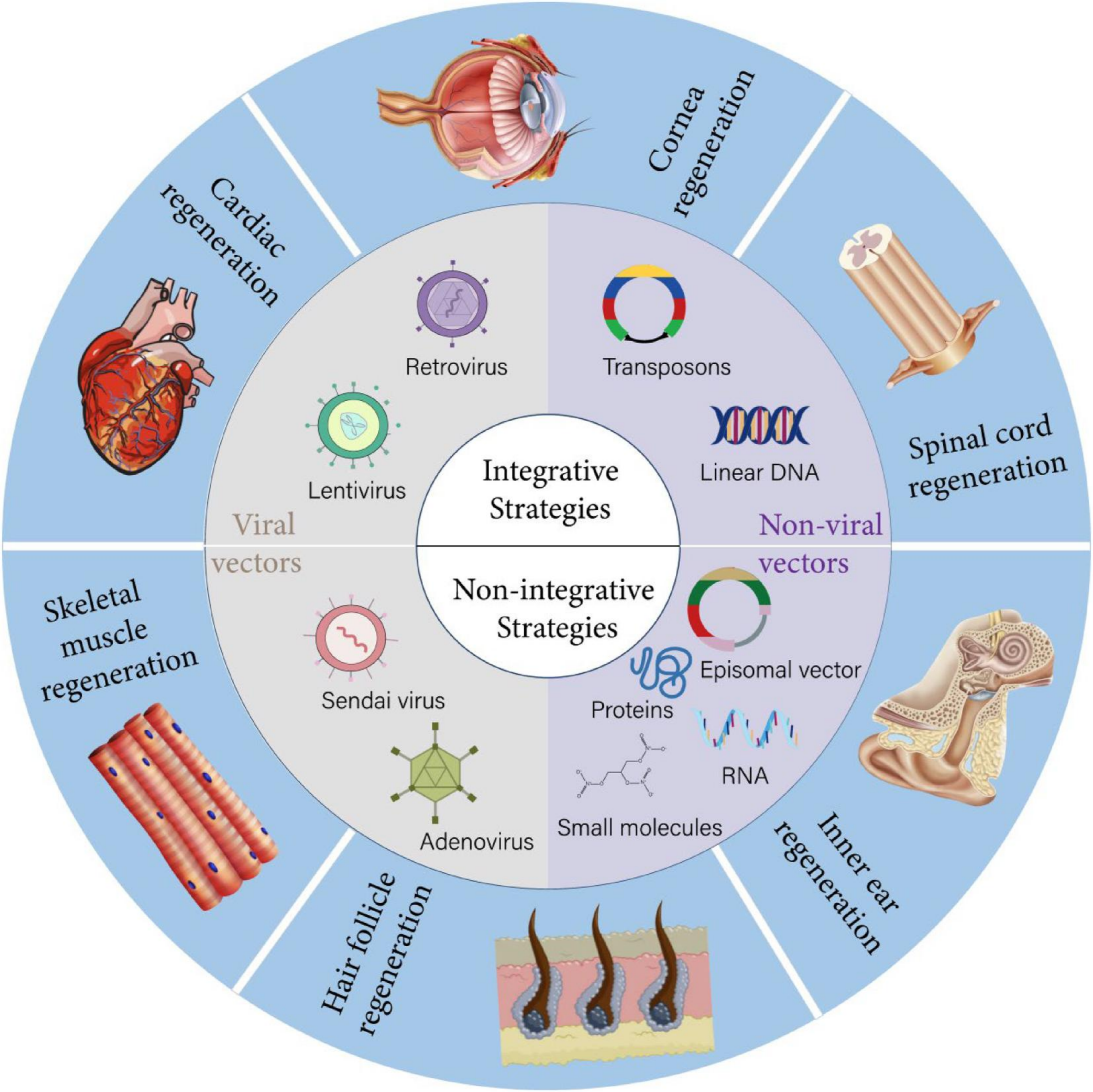
Methods for reprogramming somatic cells to iPS cells and their clinical applications

The generation of a reliable, uniform, and safe clinical‐grade iPSC population is necessary to implement future clinical trials in cell therapy. Through step‐by‐step optimization by researchers, large‐scale manufacturing of iPSCs sufficient for clinical studies is progressively achieved.^5^ The employment of reprogrammed iPSC in biomedical works can provide an important basis for an in‐depth analysis of the mechanisms of cellular reprogramming and the possibilities of reprogramming in clinical translation and medical applications.^6^ We summarize and synthesize current cutting‐edge approaches for iPSC induction and application in this review, discussing recent advances in differentiating cells such as cardiac, neural, and skeletal muscle cells from iPSCs and directly reprogramming somatic cells in tissue regeneration applications (Figure 1). Methods for delivering cells to diseased tissues are also discussed, and how these advances have contributed to advances in tissue engineering techniques for treating related disorders. Perhaps 1 day those iPSCs could be used for cell replacement therapy.

## 3 CELLULAR REPROGRAMMING

### 3.1 Definition of reprogramming

Reconditioned stem cells, alternatively called iPSC, introduce some transcribed genes into mature somatic cells of humans or animals, or chemically and physically reform adult somatic cells into pluripotent stem cells.^7^ The conventional wisdom is that fertilized ovum gradually develops and differentiates into mature cells of different organ tissues, such as bone cells, skin cells, and nerve cells, during the organism's development, while mature cells cannot reverse differentiation. Recent studies have shown that genetic modification can allow mature somatic cells to be reconstituted to form iPS cells with ESC‐like properties. iPS technology avoids the controversy of using embryos to obtain stem cells and is of great significance in advancing research on stem cells and regenerative medicine.

### 3.2 Reprogramming factor

With the help of 20 reprogrammable factors, first experiments with reprogrammed cells were then decreased to four reprogrammable factors, Oct4, Sox2, c‐Myc, and KLF‐4 (OSMK), which are known as “Yamanaka factors” and make up the bioengineering tools for transforming maturated cells into PSCs.^8^ Overexpression of reprogramming factors results in the transformation of cells into a pluripotent state. Furthermore, Nanog, OSMK, and Lin28 are often present as reprogramming factors during reprogramming. Reprogramming factors are available in various permutations with Oct4, Sox2, and Nanog forming a feed‐forward loop involving the expression of a minimum of 353 protein‐coding genes plus two miRNA genes.

### 3.3 Reprogramming cells

**FIGURE 2:**
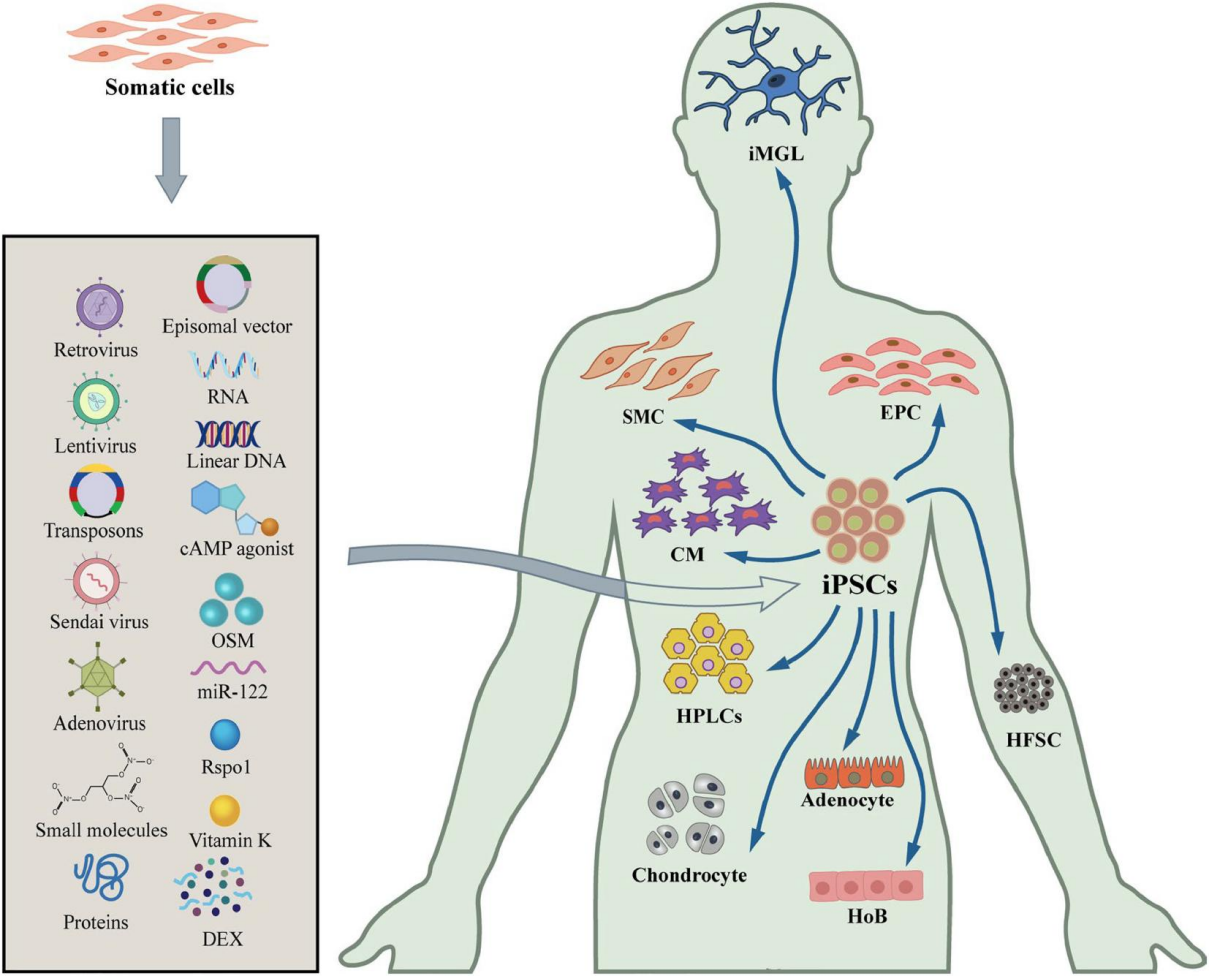
Methods of generating induced pluripotent stem cells and their applications in biology

Reprogramming is the process of reprogramming any somatic cells to iPSC with the help of reprogramming factors, and recent studies have shown that physicochemical properties can also reprogram body cells into PSCs.^9^ Progenitor cells frequently involved in reprogramming are fibroblasts, neuronal cells, keratin‐forming cells, adipose stromal cells, bone marrow mesenchymal cells, and dental pulp cells (Figure 2). Yet, the combination of reprogramming factors, method of delivery, source of somatic cells, as well as conditions of cell culture has to be considered in the reprogramming process.^1^

## 4 BIO‐DELIVERY METHODS FOR REPROGRAMMING FACTORS

Analysis of microarrays demonstrated the existence of classic stem cell gene exposure patterns among almost all iPSC clones. The utilization of various reprogramming approaches was comparable and no significant differences were found between reprogramming approaches affecting gene expression profiles comparable to that of the derived human iPSC.^10^

### 4.1 Retrovirus

The first vectors that were used as reprogramming factors were retroviruses. The retroviral vectors, mainly derived from HIV, can deliver 8–10 kb transgenes. First, iPSC was induced using Moloney mouse leukemia virus (MMLV) derivative retroviral vectors, which can deliver as much as 8 kb of the transgene, although they can only enter dividing cells.^11^ The infection productivity of iPSC cells is approximately 90% with the use of MMLV‐derived vectors, while the production yield ranges from 0.01% to 0.1%. Transcriptional vectors are integrated into host cells' genomes and drive the exposure of multipotency factors. Allosteric production of pluripotency factors converts mature cells to a pluripotent condition, resulting in the silencing of the retroviral transgene.^12^ Retroviral transduction has been effectively used to reprogram mouse and human keratin‐forming cells, neural stem cells, fibroblasts, adipocytes, as well as other cells. This reprogramming method is relatively inefficient but more efficient than when plasmid vectors are used. However, the major drawbacks of delivery of viral‐associated factors to the cellular genome are the appearance of genomic destabilization, mutations, and potential for cellular malignancy due to the integration of bulky viral constructs to the genome, involving tumorigenicity of the transcription factors delivered.

### 4.2 Lentivirus

Lentiviruses have a higher reprogramming efficiency compared to retroviruses. Rivetti di Val Cervo et al.^13^ demonstrated a method to be possible to generate from human astrocytes by in vitro overexpression of three transcription factors Lmx1a, Ascl1, and NeuroD1, and microRNA miR‐218 in a lentiviral vector induced dopaminergic neurons, a reprogramming efficiency of up to 16%, which can be used to treat neurological disorders like Parkinson's (PD). In addition, retroviral transduction is entirely ineffective for nondividing cells, such as neurons. Lentiviral vectors were more efficient in delivering gene constructs for slow‐divided cells like neurons. Morita et al.^14^ demonstrated that reprogrammed EC could be used in implantable vascular networks; however, although these reprogrammed cells exhibit high levels of expression of clear endothelial cell markers, they do not express NOS_3_, but produce nitric oxide in adult endothelial cells, which is essential for vascular function. Although the simplicity, high infection efficiency, and the ability to infect dividing and nondividing cells make this approach to gene therapy for cell replacement by lentiviral reprogramming in vivo very appealing, numerous issues are yet to be resolved, such as the risk of genomic integration and mutagenesis associated with this approach, and it is still relatively low reprogramming efficiency. Therefore, we must be cautiously optimistic about the role of lentiviral reprogramming in the future development of regenerative medicine.^15^

### 4.3 Transposon carrier

Unique DNA elements named transposons are which may be incorporated into or excised from chromosomes. Different nonviral transposons have been developed to induce cell pluripotency and directed differentiation into desired systemic cell types, and transposons acquire reprogramming efficiency higher than other reported nonintegrating delivery systems.^16^ PiggyBac, a transposon with high transduction efficiency and loading capacity, allows for precise excision of integrated transgenes with the existence of transposase. That property allows piggybac vectors for efficient transgene expression as well as a free‐transgene state, which makes a promising approach for cellular reprogramming. Katayama et al.^17^ attempted to produce iHeps using the piggyBac transposon system to integrate transgenes composed of Foxa3 and Hnf4a and succeeded in obtaining functional iHeps. They then demonstrated the removal of transgenes for obtaining transgene‐free iHeps, and it still maintains the hepatocyte function. Such a nonviral, transgene‐free reprogramming approach with piggyBac vectors could promote cellular therapies for iHeps in forthcoming clinical applications. Similarly, Luo et al.^18^ produced a PiggyBac transposon vector to achieve co‐expression of buffalo Yamanaka factors genes (PB_OMKS) on buffalo fetal skin fibroblasts (BFSF) to establish the BFSF_OMKS cell line, and their results showed that the overexpression of Yamanaka factors leads to the activation of reprogramming‐related LIF, activin, BMP4, SMAD1/5/9, and Wnt signaling. Such outcomes increase our insight into the mechanisms of reprogramming in buffalo somatic cells and may offer potential theories for the selection of blends of small molecules to facilitate reprogramming. In addition to piggyBac, the Sleeping Beauty (SB) transposon represents an excellent tool for advanced genetic engineering and a valuable module for studying DNA transposition in vertebrate cells. Kesselring et al.^19^ identified novel SB transposase mutants and established compensatory amino acid substitutions that could completely save these mutants from integration defects, showing that shearing and pasting DNA transposition could be transformed into a unidirectional procedure by individual amino acid shifts.

### 4.4 Adenovirus

In recent years, in direct reprogramming techniques, somatic cells may be induced to achieve the reprogramming of PSC through the overexpression of specifically identified factors; among the variety of methods used to transport genes to cells, the genomic integration viral vectors commonly used may lead to undesirable events such as integration‐related mutagenesis, making integrative viral reprogramming vectors unsuitable for clinical applications.^11^ Nonintegrating viral vectors do not risk genomic integration, but the reprogramming efficiency of nonintegrating viral vectors tends to be low at approximately 0.02%. Adenoviral vectors are nonintegrating viral vectors capable of producing transgene‐free iPSC, and this reprogramming method has a low oncogenicity rate. However, reprogramming by adenoviral vectors is an inefficient approach that requires high titers of virus and sometimes even repetitive transduction. By forcibly expressing OSKM, somatic cells can be temporarily reprogrammed to a multiplying, de‐differentiated state by forcibly OSKM. Kisby et al.^20^ presented evidence for the first time in which adenoviral OSKM delivery may lead to partially reprogrammed postnatal cardiomyocytes to induce transient reprogramming of mammalian heart myocytes using a nonintegrating vector encoding OSKM in vitro. Following induction, expression of reprogramming factors in mouse and rat cardiac myocytes provoked fast but restricted cellular dedifferentiation. Concurrently, enhanced gene expression increased Ki67‐positive cells, and enhanced cell cycle activation associated with cell cycling was observed. Such transient property of some reprogramming was shown to occur on day 15 after cardiomyocyte‐specific viral transduction with the spontaneous recovery of cell shape, expression of genes, and activity of contraction. Thus, the transient adenovirus‐mediated reprogramming may be a new and feasible mechanism of regeneration.

### 4.5 Sendai virus

Sendai virus vectors (SeVdp) are the most popular and safest method for obtaining iPSC based on viruses. As they are regarded as “zero‐footprint,” that is, not entering the nucleus, they duplicate within the cytoplasm of the affected cells without integrating into the genome of the host cell.^21^ Inducible pluripotent stem cells can be generated with Sendai virus vectors with very high efficiency, allowing the delivery of up to four exogenous genes in a vector with a reprogramming efficiency of about 1%, stable and reproducible reprogramming. These vectors have a wide range of tropism and allow transduction of many types of cells and thus have multiple uses. Human PSCs can potentially diverge into a wide diversity of cell types, among them skeletal muscle (SkM), which are used in regenerative medication or modeling of refractory diseases in vitro. Tan et al.^22^ established a simple differentiation method to accelerate the study of neuromuscular diseases by transforming human PSC into SKM cells expressing SKM markers, including the myogenic master transcription factor MHC, using a temperature‐sensitive Sendai virus‐encoded myogenic determinist 1 (SeV‐Myod1) (SeV) vector. They then performed a temporary treatment at 38°C to remove the SeV vector, which additionally speeds up the differentiation of the mesoderm and reveals that SkM cells exhibit a fibrous morphology. Eventually, following the targeted delivery of cytotoxic compounds by pluripotent stem cells to remove residual SeV vectors, they produced SkM cells with 80% MHC positivity and responding to electric stimulation. Such a simple myogenic differentiation method applies to human iPSC and will facilitate future research on disease mechanisms and drug discovery.

### 4.6 Minicircle DNA

iPSCs derived from samples of patients have great possibilities for disease pathology studies and therapeutic regenerative medicine. Nevertheless, the majority of iPSC‐derived technologies employs integrating viruses, and this may depart residual transgenic traces, which are part of the host genome, thus unpredictably altering the phenotype of cells in downstream applications. Narsinh et al.^23^ described a protocol for constructing minicircle DNA into human ASCs by transfection of a simple nonviral derived iPSC. The minicircle DNA vector does not contain viral DNA and can be highly expressed in mammalian cells. Minicircle DNA has great potential for pathology studies, but it can also reprogram cancer cells toward iPSCs or weaker invasive cancer cells to research cancer‐related genes as well as their interaction with the cellular environment before and after reprogramming. For example, Câmara et al.^24^ designed to study the ability of mouse melanoma B16F10 cells to reprogram. They first demonstrated that B16F10 reprogramming could be triggered with nonviral minicircle DNA containing four reprogramming factors Oct4, Sox2, Lin 28, Nanog (OSLN), and GFP reporter genes by IF, RT‐PCR analysis, and cell cycle results. This results in clones composed of epithelioid cells that exhibit cancer stem cell characteristics, thus exhibiting PSC markers and undergoing asymmetric or symmetric division. Reprogrammed B16F10 cells cannot form teratomas, and compared to the parental B16F10 cell line, they exhibit tumor‐suppressive capacity characterized by a reduction in tumor size. The results suggest that it is possible to obtain reprogrammed cancer cells with lower invasiveness in mouse melanoma due to the reprogramming of B16F10 cells. Such cells present a fascinating template for the research of cellular malignancy mechanisms and offer a new instrument for anticancer drug filtering.

### 4.7 Episomal vectors

Reprogramming using nonintegrating exosomal vectors is a convenient, safe, and cost‐effective method that has emerged as an important instrument for inducing PSC reprogramming. Exosomal vectors can generate iPSCs without exogenous DNA induction. Exosomal vectors harboring “Yamanaka reprogramming factors” are important vehicles for reprogramming cells to a pluripotent condition without integration. Nevertheless, the reprogramming procedure is still largely stochastic and is hindered by the fact that exosome‐carrying vectors cannot be easily and rapidly cloned. Schmitt et al.^25^ adapted the primary vectors to enrich transfected cells by expressing spectrally separable fluorescent proteins. They were then tested against the modeled original vectors to see if they could be effectively reprogrammed. Recombinant vectors permit cell sorts according to the expression of reprogramming factors, can help track the exosome expression of individual cells, and allow the selection of the dose of reprogramming factors. These improved vectors facilitate the understanding of the reprogramming process and increase the extraction efficiency of iPSCs. In addition to the inability to identify carrying episomal vectors easily, genomic integration of exogenous genes is occasionally observed as a problem with episomal vectors. Furthermore, removing free DNA in established iPSCs takes more than 70 days. Lee et al.^26^ inserted cytosine deaminase (CD) genes from yeast into an exosome vector and utilized them for reprogramming human fibroblasts into iPSCs. These CD exosomal vectors could be removed from the produced iPSCs just 7 days after 5‐fluorocytosine (5‐FC) therapy. They further discovered that integrated CD gene cells died in 2 days of 5‐FC treatment. Furthermore, they produced neural stem cells without exogenous integration by directly reprogramming CD exosomal vectors in combination with 5‐FC treatment. In conclusion, this new CD‐free vector allows for quick and convenient isolation of reprogrammed cells without exogenous integration and could be used for disorder modeling and medical applications.

### 4.8 RNA

RNA provides two inherent advantages; they do not integrate into the genome and do not require its transport to the nucleus. Thus, RNA can be an alternative strategy to be applied for reprogramming. The delivery of synthesized mRNA directly into body cells to elicit pluripotency is a strategy to generate iPSCs with the least retained footprint and genomic integration. Furthermore, RNA delivery offers the greatest reprogramming productivity versus other nonviral nonintegrated delivery methods, and is, therefore, the more promising approach for future medical applications. Feng et al.^27^ constructed Linc‐ROR lentivirus‐mediated B in vitro microcultures as well as in vivo MeHA hydrogel wraps, respectively. Linc‐ROR‐related miRNAs that inhibited SOX9 exposure were detected via luciferase testing, real‐time PCR together with protein blotting. Ectopic expression of linc‐ROR markedly facilitated chondrogenesis in BMSCs in vitro and chondrogenic activity in vivo. linc‐ROR regulates chondrogenic differentiation and chondrogenesis in BMSCs by serving as a competitive internal RNA for miR‐145 and miR‐138, and activating SOX9. Therefore, Linc‐ROR can be regarded as a novel diagnosis and treatment target for the treatment of OA. Numerous studies showed that microRNAs could improve the reprogramming productivity of iPSC. Zhang et al.^28^ in a lentiviral system driven by tetracycline (TET) carrying OSKMNLST to reprogram HEK293FT into PSCs, found that the overexpression of miR‐200c‐141 in combination with OSKMNLST remarkably increased the productivity of sheep iPSC generation, and these cells showed pluripotent properties similar to ESC, were alkaline phosphatase and pluripotency marked by qRT‐PCR and IF, and were positive able to differentiate in vitro into three germ layers. A dual‐luciferase reporter assay was used to target the zinc finger E‐box binding homology box 1 (ZEB1) 3′UTR. qRT‐PCR and WB revealed that the overexpression of miR‐200c‐141 significantly reduced the ZEB1 expression but increased the E‐calmodulin expression. This indicates that miR‐200c‐141 promotes sheep body cell reprogramming, which may be achieved by reducing the expression of ZEB1 3′UTR and increasing the expression of E‐calmodulin.

### 4.9 Protein

Conventional iPSC reprogramming methods suffer from insertional mutagenesis due to integrated viral vectors. Although some progress has been achieved in alleviating this problem, involving the usage of small chemical molecules, as well as excisable and nonintegrating usage of modified mRNA, security is still an issue. Integrated and nonintegrated approaches are also limited by inefficiencies, mutability, and oncogenicity. Nonintegrated mRNA reprogramming is effective yet sensitive to reagents, requiring methods that reduce immunogenic responses. Another nonintegrated and safer method of generating iPSC is by reprogramming recombinant cells directly to the cell to reprogram, although protein‐based methods are less efficient at reprogramming than traditional virus‐based nuclear reprogramming.^29^ Cho et al.^30^ described a new method for the delivery of reprogrammed proteins into differentiated fibroblasts with titanium dioxide (TiO_2_) nanotubes, which are free of cytotoxic effects as well as do not impair cell proliferation. Following treatment with protein‐bound nanotubes for 3 weeks, the somatic cells adopt an ESC‐like shape and express activated Oct4‐green fluorescent protein, a pluripotent biological marker. The results indicate that titanium dioxide nanotubes were employed to deliver reprogramming factors directly to somatic cells to induce pluripotency. Aiming to overcome the tumorigenic risk associated with pluripotent and pluripotent stem cells, Yang et al.^31^ previously established a new reprogramming technique to generate pluripotent units out of skin fibroblasts with a single protein, fibronectin (FMOD), and its product. FMOD reprogrammed (FReP) cells, which exhibit excellent myogenic capacity without the risk of tumorigenicity, are a hopeful and secure source for the establishment of skeletal muscle. Besides insertion with the best cells, maintaining cellular positioning and preservation in the receiving tissue environment is equally important for establishing muscle tissue of critical dimensions. Thus, they demonstrated that photopolymerizable methacrylate‐glycolylated chitosan (MeGC)/collagen type I (ColI)‐hydrogels provide an ideal three‐dimensional microenvironment for the survival, extension, spreading, and myotube generation of FReP cells encapsulated within the hydrogel. In vitro, FReP cells did not differentiate in undesired directions, such as osteogenesis, cartilage, or tendon. Furthermore, the genetic analysis revealed paired box 7 (PAX7) → myogenic factor 5 (MYF5) → myogenic assay 1 (MYOD1) → myogenic protein (MYOG) → elevated myosin cassette in FReP cells encapsulated during myogenic differentiation, indicating that the FReP cell‐MeGC/ColI‐hydrogel construct is a hopeful tissue engineering mimic for in vitro skeletal myogenic subjects and hence has exceptional possibility for further in vivo study validation.

### 4.10 Liposomal magnetofection

Magnetic infection of liposomes is a technique for nonviral delivery of nucleic acids to cultivated cells. For this technique, self‐assembled compounds of enhancers, such as nucleic acids and cationic lipids, are mixed by magnetic nanoparticles and then focused on the cell surface by applying a permanently magnetic field for highly efficacious delivery. Park et al.^32^ introduced a liposome magnetic infection (LMF) method to generate iPSC. By using various levels of CombiMag‐DNA nanoparticles (pCX‐OKS‐2A and pCX‐cMyc) liposomes with various mixtures and one or two cycles of the LMF process, effective virus‐free iPSC generation from mouse embryonic fibroblasts (MEF) was determined conditions. The reprogramming time was short (≤8 days, 0.032%–0.040%). Of the tested seven LMF‐iPS cell lines, both were found to be absent of integration as well as producing integration‐free LMF‐iPS cell lines with minimal toxicity conditions (half‐dose plasmid with a single LMF cycle). Cells of this cell clone showed also in vivo/in vivo pluripotency involving the formation of teratomas and the generation of chimeric mice. Furthermore, the security of CombiMag‐DNA liposome‐transduced MEF cells was verified by assay of lactate dehydrogenase activity and transmission electron microscopy. LMF possibly represents a brilliant technique for generating nonviral or nonintegrated iPS cell lines, which will help to strengthen stem cell therapies in the future.

## 5 MODULATION OF REPROGRAMMING BY CHEMICAL FACTORS

**FIGURE 3:**
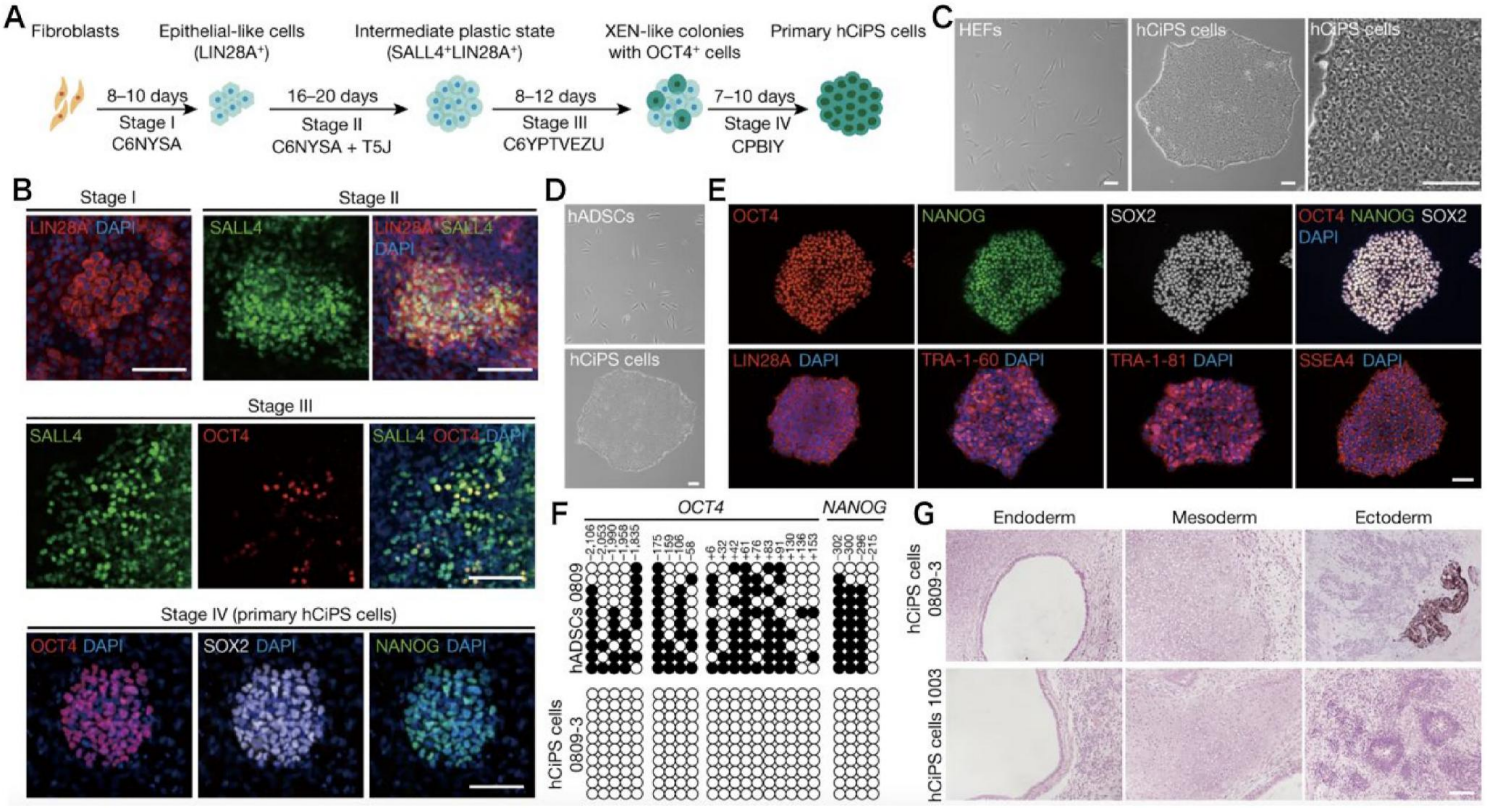
Chemical reprogramming of fibroblasts into hCiPS cells by chemical methods. (A) Schematic representation of chemical reprogramming from fibroblasts into hCiPS cells. (B) Immunofluorescence analysis of pluripotency‐associated genes at the end of stages I, II, III, and IV. (C) Morphology of human embryonic fibroblasts (HEF) and HEF‐derived hCiPS cells. (D) Morphology of hADSCs and hADSC‐derived hCiPS cells. (E) Immunofluorescence analysis of pluripotency markers in hADSC‐derived hCiPS cells. (F) Bisulfite sequencing of DNA CpG methylation status of OCT4 and NANOG to analyze promoter motifs in hADSCs and hADSC‐derived hCiPS cells. (G) Representative images of hematoxylin and eosin staining showing endoderm (respiratory epithelium), mesoderm (cartilage), and ectoderm (pigmented retinal epithelium and neural tissue) of a single teratoma from the hCiPS cell clone shown.

Somatic cells can be reprogrammed to a pluripotent stem cell (CiPSC) state by chemical stimulation (Figure 3). Hou et al.^33^ showed that it is possible to reverse cell fate and reprogram mouse somatic cells into chemically iPSCs (CiPS cells) using only exogenous chemical small molecules without relying on endogenous cellular substances, such as oocytes and transcription factors. Compared with traditional methods, chemical small molecule manipulation is simple and flexible, with spatiotemporal solid regulation and reversible effects, allowing precise manipulation of the cell reprogramming process. In addition, small molecule‐induced somatic cell reprogramming is a nonintegration method that avoids the safety issues associated with traditional transgenic manipulation and is expected to become a safer clinical treatment. Yang et al.^34^ found that the application of both CHIR99021 (a GSK3 inhibitor), 616452 (Repsox, an ALK5 inhibitor), and trichothecene (a cAMP agonist), as well as their combinations, to iPSC generation, is often used as a direct way to induce different cell types. This indicates that several common processes underlie the reprogramming of these chemically induced cell types. These chemical mixtures initially activate the mouse fibroblast in a plastic state, which then transforms into extra‐embryonic endoderm (XEN). The plasticity state is characterized by extensive activation of developmentally relevant transcription factor (TF) expression, like Tbx3, Ascl1, Sox17, and Nkx6‐1, and greater accessibility of the chromatin state, suggesting an improved capacity for cell fate transition, a state that enhances the potential for cellular adaptation to alternative cell fates. Guan et al.^35^ found that the JNK pathway is an important obstacle to chemical reprogramming and its inhibition is essential for the induction of cellular plasticity and regenerative‐like programs through the inhibition of pro‐inflammatory pathways. For inducing de‐differentiation of human somatic cells, they identified small molecules that disrupt fibroblast properties, promote proliferation, and reactivate de‐differentiation‐related gene, and identified a combination of small molecules (616452, CHIR99021, and TTNPB) that convert human fibroblasts into epithelial cells. Extra screening of the chemical library revealed that ABT869, Y27632, and SAG provided further promotion of epithelial cell generation (phase I conditions). Coherently, such treatment upregulated epithelial cell‐related genes (KRT8, KRT19, and KRT18) and downregulated a group of fibroblast marker genes, indicating a loss of fibroblast properties. Immunofluorescence and RT‐qPCR analyses indicated the expression of LIN28A in cultures, an important gene‐regulating dedifferentiation, and regeneration in different species. In addition, the chemical composition of the biological material may affect the efficiency of reprogramming. The study of which constituent materials may be appropriate to enhance the efficiency of reprogramming, Kim et al.^36^ used gelatin, hyaluronic acid (HA), chitosan, alginate, type I collagen (Col I), laminin, or fibronectin to coat cell culture in plates. Mouse embryonic stem cells (OG‐mESCs) expressing OCT4‐GFP were cultivated on these plates. OG‐mESCs were cultured on various media, and cells cultured on Col I had maximum OCT4‐GFP expression. However, their dome‐like shape morphology also became flattened. Thus, according to this result, they concluded that HA is the most helpful biomaterial for improving the efficiency of 3D reprogramming.

## 6 MODULATION OF REPROGRAMMING BY PHYSICAL FACTORS

Under physiological conditions, cells undergo physical and biochemical stimuli from their microenvironment. Two‐dimensional cell culture conditions covering a variety of biochemical cues have been extensively studied. For example, multiple viral, DNA, or RNA vectors containing multiple reprogramming factors, as well as protein or minor molecule complements in culture conditions have been used, while the impact of three‐dimensional microenvironments covering biophysical cues on reprogramming is not yet sufficiently explored. Not only are cells influenced by biochemical signals like small molecules and growth factors, but physical sensors like matrix stiffness, morphology, and coarseness also have critical effects in mediating stem cell self‐renewal, stem cell stemness and pluripotency, stem cell differentiation, and cell reprogramming. For example, stem cells acquire “mechanical memory” in the rigid physical environment in which they are cultured, thereby influencing cell fate. While many cells may be cultivated in three‐dimensional conditions, few studies concern reprogramming in a three‐dimensional microenvironment.

### 6.1 Regulation of cell reprogramming by three‐dimensional culture

Somatic cells may be reprogrammed into iPSCs, and it holds great promise for applications in regenerative medicine. Nevertheless, challenges to the successful application of human iPSCs for healthcare purposes are low generation efficiency, hesogeneous cell populations, and exposed to the animal‐derived product Matrigel. Sun et al.^37^ intended to study whether human urine cells can be reprogrammed efficiently into iPSCs in 3D‐Puramatrix in contrast to 2D‐Matrigel and to see whether this 3D hydrogel environment influences the reprogramming procedure. Interestingly, although the colonization rate in 3D‐PM was the same as in 2D‐MG (∼0.05%), the reprogrammed cells in 3D‐PM included a significantly more homogeneous iPSC population, supported by pluripotent body‐like morphology, marker expression, and transcriptome analysis at the bulk and single‐cell levels. This is evidenced by pluripotent‐like morphology, marker expression, and transcriptome analysis at the somatic and single‐cell levels. In addition, this work reveals that modulation of human somatic cell reprogramming through the extracellular microenvironment, that is, increased homogeneity of the iPSC population in 3D‐PM colonies is associated with downregulation of ITG‐β1 and phosphorylated adhesion site kinase (FAK). Overall, 3D‐PM offers an additional method to obtain iPSC with enhanced homogeneity.

### 6.2 Regulation of cellular reprogramming by stiffness

Previous studies have demonstrated that the efficiency of reprogramming could be regulated by adjusting the hydrogel's surface hardness. With softening of the hydrogel, the cells exhibit a more circular morphology with a tendency to acquire more mesenchymal‐epithelial transition (MET), which is the first step in iPSC reprogramming, thus increasing the efficiency of reprogramming.^38^ To determine the role of physical signals in inducing cell reprogramming, Choi et al.^39^ produced cell culture matrices of different hardnesses (0.1, 1, 4, 10, and 20 kPa) using polyacrylamide (PA) gels. To assess whether the physical signal of the substrate affects reprogramming efficiency, they knocked down OSKM‐transduced Oct4‐green fluorescent protein (GFP) into MEF (Oct4‐GFP MEF). They inoculated them on hardness‐controlled hydrogel substrates that were coated with the same gelatin density. To quantify reprogramming efficiency by counting the number of Oct4^−^GFP^+^ colonies 21 days after transduction, Oct4^−^GFP^+^ colonies of MEFs precultured on 0.1 kPa hydrogel substrates were more than twofold higher than on harder hydrogel matrices (1–20 kPa). This difference in reprogramming efficiency versus substrate stiffness suggests that cell reprogramming is promoted on softer hydrogels. Kim et al.^36^ also assessed the influence of hydrogel modulus on reprogramming to iPSC. To begin with, MAHA hydrogels with different moduli were fabricated by taking control of the degree of substitution (DS) of MA. They then wrapped mouse embryonic stem cells (OG‐MEF) with these hydrogels to reprogram them into iPSC. The shear modulus of MAHA was 109.71 ± 19.88 Pa, 66.21 ± 11.76 Pa, 50.00 ± 10.59 Pa, and 10.11 ± 0.42 Pa in DS100, DS80, DS50, and DS10, respectively. They then evaluated the OG‐MEF ratio for reprogramming into OCT4‐GFP+ iPSC cells from these different MAHA hydrogels. Ultimately, it was discovered that reducing the MAHA modulus markedly improved the efficiency of reprogramming to iPSC by downregulating OCT4 and NANOG.

### 6.3 Regulation of cellular reprogramming by surface morphology

**FIGURE 4:**
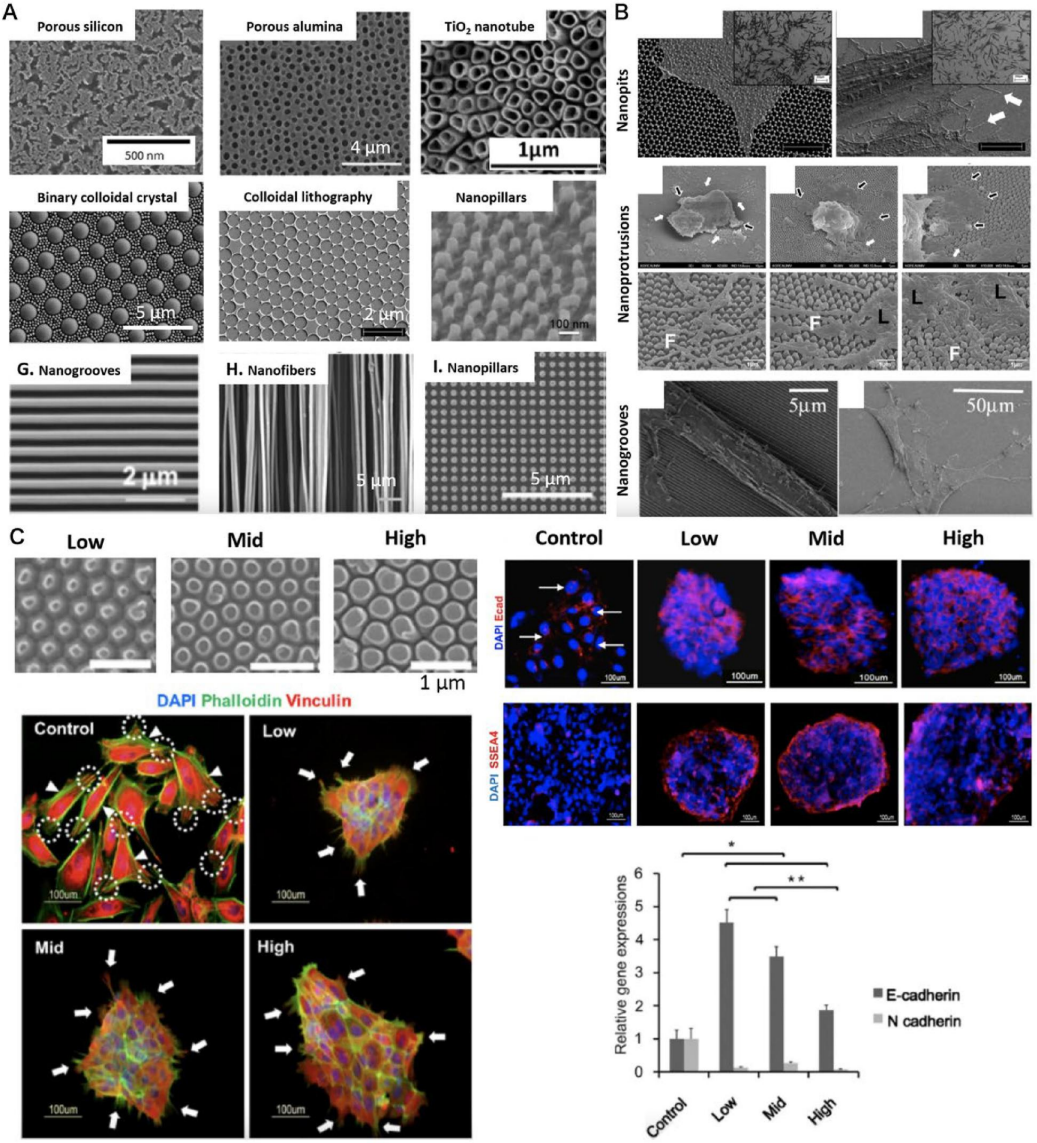
Effect of surface morphology on cellular reprogramming. (A) Nanomorphology on different materials fabricated by different methods. Binary colloidal crystals (BCC) are composed of porous silicon (pSi), porous alumina (pAl), TiO_2_ nanotubes, 2 μm Si particles, and 200 nm PS. Colloidal lithography patterns (tantalum), nanopillars (titanium), nano grooves (urethane acrylate), and electrospun nanofibers (chitosan). (B) Stem cell growth on different nano topologies: Fewer filamentous pseudopods and higher osteogenic differentiation of human adipose stem cells (hADSCs) were found on nanoporous surfaces with pore diameters of ∼700 nm compared to planar controls. Smaller cell areas and higher OCT4 expression were found for human embryonic stem cells (H9) on polystyrene nanopillars than in planar controls. The larger the size, the higher the filamentous pseudopod/lamellar pseudopod ratio. Aligned hMSC cell morphology and lower proliferation were found on the nanogroove surface than planar controls. (C) Nanopodia maintains the pluripotency of human ESC (H9). Nanoprompters were made on PS of different sizes: low (diameter 120–170 nm; spacing ∼300 nm), medium (diameter 170–290 nm; spacing ∼200 nm), and high (diameter 290–360 nm; spacing ∼100 nm). Immunostaining showed that colonies cultured on nanopatterns expressed higher levels of E‐calmodulin (cell‐cell contact) and SSEA4 (undifferentiated marker) than the control. Immunostaining showed that the colonies cultured on the nanopattern had a 3D‐like structure than the control. In addition, the adherent spots (nucleoproteins) were more prominent on the control than those on the nanopattern. Gene expression of E‐ and N‐calmodulin suggests that hESCs on the control are undergoing an epithelial‐mesenchymal transition (EMT)‐like process.

The interaction of stem cells with the ECM is critical for stem cell self‐renewal and polarization, where surface nanomorphology is an important factor in controlling stem cell responses, including cell therapy, cell culture, and regenerative medicine applications (Figure 4). Novel methods have emerged to involve the use of advanced manufacturing surface immobilization approaches, and iPSCs are beginning to be studied around the interactions of the cell culture medium's surface properties and the cell culture medium's biomolecular composition.^40^ All these influence the stem cell response and determine the exact effect that each factor plays, and the synergy between these factors is critical for future development. Carson et al.^41^ proposed the use of nanogroove lattices composed of self‐assembled chimeric peptides (PUABP2‐RGD) with groove widths ranging from 350 to 2000 nm laid on the surface to investigate the influence of various nanoscale structures on the development of the structure of human iPSCs‐derived cardiomyocytes. They found that cardiomyocyte organization and structural product in a biphasic manner depended on the nanotopographic feature size, achieving better growth on grooves ranging from 700 to 1000 nm. They found that the ideal width range for inducing cardiomyocyte development exists in the 30–120 nm range relying on observations of in vivo oriented ECM fibers. Although they both fall within the nanometer range, a comparative review of in vitro and in vivo data indicates that a broader topology is required in vitro to promote cardiomyocyte maturation. This difference may be ascribed to the inherent discrepancy between the three‐dimensional and two‐dimensional microenvironments. In three dimensions, cardiomyocytes are enclosed and compressed by ECM fibers and other axial cells. As a result, they are more susceptible to the mechanical cues that surround them. Conversely, in two dimensions, the cells in the culture are less mature and are only affected by topographical cues in a unique plane. Thus, these cells may need significantly more distinct structural clues to direct their uniaxial growth and maturation. Such discoveries underscore the ability of surface topography to influence cardiomyocyte development and the ability of this approach to investigate the role of topographic cues on cell behavior as a general assay.

## 7 CELLULAR REPROGRAMMING IN BIOMEDICAL ENGINEERING

The discovery of iPSCs has been a precious contribution to regenerative medicine, which paved the way for determining the true human embryonic stem cell (ESCs) potential. As the ethical controversy surrounding ESCs is still being contested, iPSCs have been used to circumvent the process of destroying human embryos.^7^ iPSCs have been widely used for hair follicle regeneration, myocardial infarction, neurological diseases, liver diseases, and spinal cord injuries. The current review focuses on presenting the translational clinical research surrounding iPSC with its growing possibilities in the clinical field.

## 8 CELLULAR REPROGRAMMING IN CORNEAL REPAIR

**FIGURE 5:**
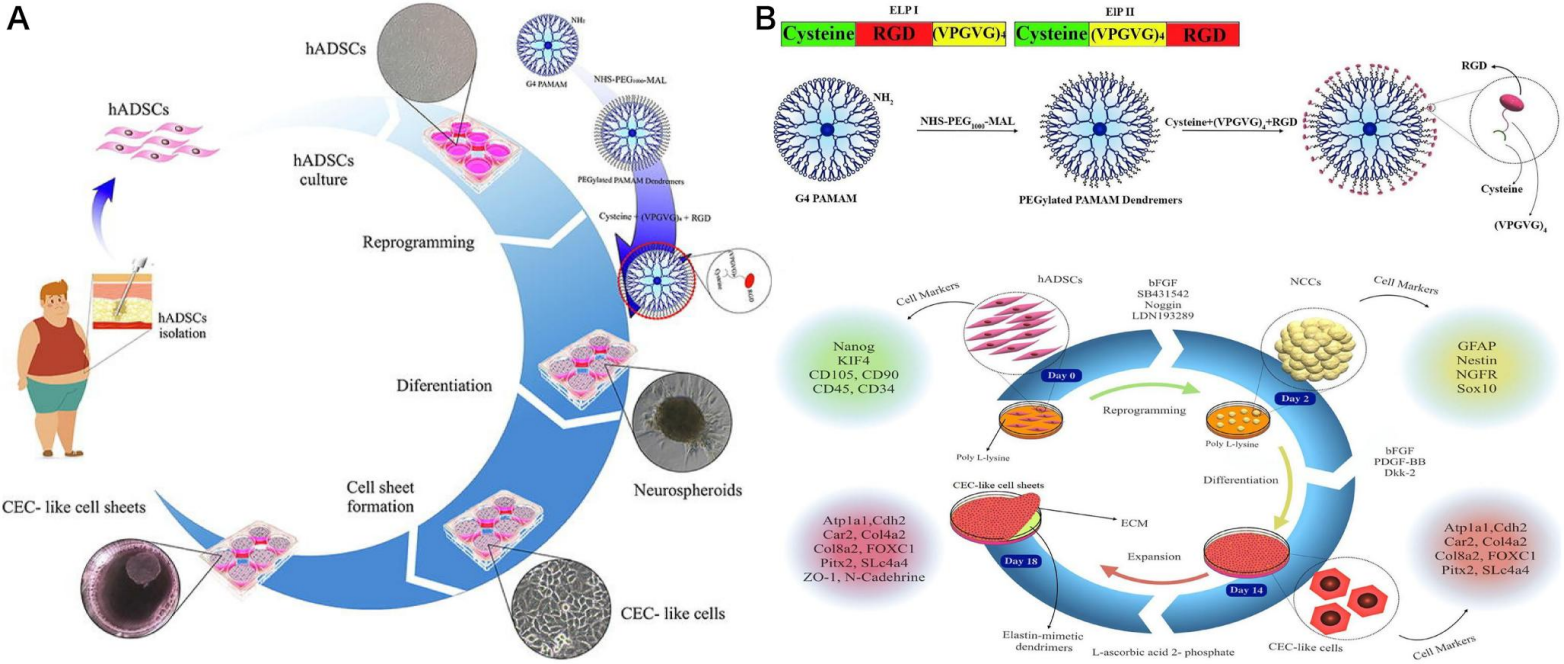
Reprogramming techniques in corneal repair (A) Graphical summary of heat‐responsive elastin‐like dendritic molecules on hADSCs isolated and differentiated from adult corneal endothelial cells (HCECs)‐like cell sheets. Hematopoietic stem cells were obtained by liposuction. There are two differentiated stages in the protocol. First, hADSCs are reprogrammed into neural spine cells (NCCs) by poly‐l‐lysine, then differentiated into CE‐like cells on dendrimer that mimic elastin and cultured until CE‐like cell sheets are formed. (B) Schematic representation of the amino acid sequences of ELP I and ELP II. Schematic diagram of the temperature‐sensitive synthesis of a mimic elastic dendrimer. hADSCs differentiate into CECS‐like cells at the stage of differentiation. Finally, CECS‐like cell sheets are formed on heat‐responsive mimic elastic dendritic molecules. Growth factors and specific markers used at each step of the differentiation protocol are shown.

The limitations of human corneal endothelial cell (HCEC) division in vivo and the shortage of corneal donations have led to an increasing interest in bioengineering corneal endothelium (CE) using replacement cells. iPSCs hold great potential for regenerative medicine. Chien et al.^42^ succeeded in reprogramming human corneal keratinocytes into iPSCs and demonstrated that iPSCs can promote corneal reconstruction. They cultured these iPSCs in a serum‐free and feeder layer‐free system, and after 30 generations of passages, these iPSCs remained stable and exhibited pluripotency like ESCs. Subsequently, they developed an injectable amphiphilic carboxymethylhexyl chitosan (CHC) nanoscale hydrogel. They discovered that this gel enhanced the viability of iPSC and the proportion of CD44^+^ cells and kept their stemness. iPSC with CHC hydrogel (iPSC/CHC hydrogel) promoted corneal abrasion wound healing. Khalili et al.^43^ fabricated thermally responsive biopolymers by combining RGD‐functionalized elastin‐like peptides (ELPs) as nanoplatforms with G4 dendrimers, called elastin‐mimetic dendrimers (EMDs) (Figure 5). The hADSCs were firstly reprogrammed into neurospheres induced by small molecules with poly‐L‐lysine. Neural crest cells (NCCs) were then differentiated into CE‐like cells on EMD and cultured until they formed CE‐like cell sheets. EMD exhibits good biocompatibility and a lower critical liquefaction temperature (LCST) close to physiological temperature, which improves cell adhesion, spreading, differentiation, and proliferation of NCC to CE‐like cells, making it desirable for CE‐like cell sheet collection. NCC and CE‐like cell markers were identified by qRT‐PCR, immunocytochemistry, and WB. 18 days later, cell sheets with cell junctions were harvested by lowering the temperature. Thus, this research successfully reprogrammed hADSC to CE‐like cells on EMD and successfully generated CE‐like cell sheets, demonstrating that cell therapy of the cornea can be accomplished with reprogrammed tissue regeneration engineering. The technology‐based on iPSC allows surgical residues, usually considered medical waste, to be used as an additional source of PSC for personalized medicine and will have a wide range of applications in the future.

## 9 CELLULAR REPROGRAMMING IN CARDIOVASCULAR REPAIR

**FIGURE 6:**
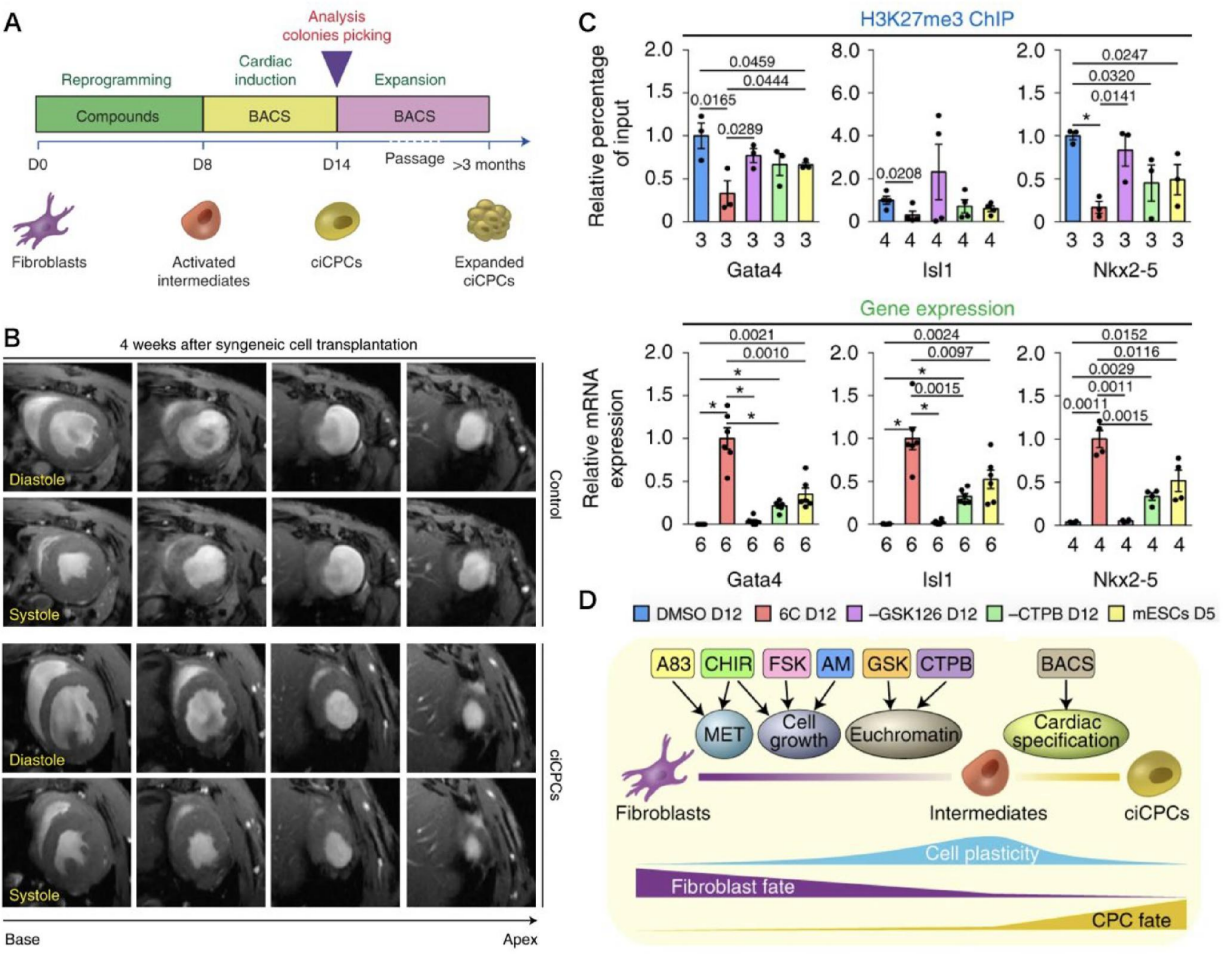
Reprogramming of fibroblasts into expandable cardiovascular progenitor cells by small molecules. (A) Schematic representation of ciCPC generation. (B) Representative T2‐weighted MRI by magnetic resonance (MRI) 4 weeks after MI in wild‐type mice receiving vector control or homozygous ciCPC transplantation. (C) ChIP‐qPCR analysis of H3K27me3 deposition and qPCR analysis of gene expression at the indicated loci (*n* = 3–6 biologically independent samples, exact n is labeled under the corresponding bar). (D) Model for 6C‐induced CPC reprogramming.

The lack of autologous stem cell sources that could be expanded over time and have true cardiovascular differentiation potential is a major obstacle to cardiac cell therapy.^44^ Human iPSCs have an unlimited ability to self‐renew and to differentiate into multiple cardiac cell types. Thus, iPSC technology holds the promise of providing cell‐based personalized medicine for patient‐specific cardiovascular repair. Wang et al.^45^ reprogrammed mouse or human fibroblasts (HDF) into proliferating cell populations with strong cardiovascular differentiation potential by a combination of six compounds (6C). These chemo‐induced cardiovascular progenitor cells (ciCPCs) are capable of long‐term self‐renewal and maintain a sustained CPC phenotype and cardiovascular differentiation capacity in vitro and in vivo (Figure 6). Implantation of ciCPCs into the hearts of infarcted mice (MI) led to improved animal survival and cardiac function and maintained mouse survival until 13 weeks post‐infarction. Among the cell sources generating iPSCs for cardiovascular repair, human peripheral blood may be more available than the already widely used human skin fibroblasts (HDF). Kim et al.^46^ successfully isolated and cultured human heart‐derived circulating cells, called circulating pluripotent stem cells (CiMS), from 10 ml of blood. They researched the generation efficiency of CiMS‐derived iPSC (CiMS‐iPSC). They studied the possibility of CiMS‐iPSC differentiating into the mesodermal lineage and cardiovascular cells like vascular smooth muscle cells, endothelial cells, and cardiomyocytes with higher efficiency compared to HDF‐iPSCs or hESCs. In addition, they examined the epigenetic status of different cell types. Although methylation of the Brachyury T promoter CpG site did not differ between cell types, histone H3 lysine 4 trimethylation levels were enhanced in the Brachyury T promoter region in CiMS‐iPSCs compared to other cell types. Conversely, histone H3 lysine 9 acetylation was downregulated during the differentiation of CiMS‐iPSCs. A myocardial infarction model more significantly different from CiMS‐iPSCs showed greater therapeutic potential for myocardial regeneration than other cell types. Peripheral blood may also show therapeutic potential in myocardial regeneration. However, applying peripheral blood‐derived circulating pluripotent stem cells requires more complex methods and steps. Based on these results, CiMS cells have the potential to be used in regenerative medicine with cell therapy.

## 10 BIOMATERIALS TO REGULATE CELL REPROGRAMMING IN NEURAL REPAIR

**FIGURE 7:**
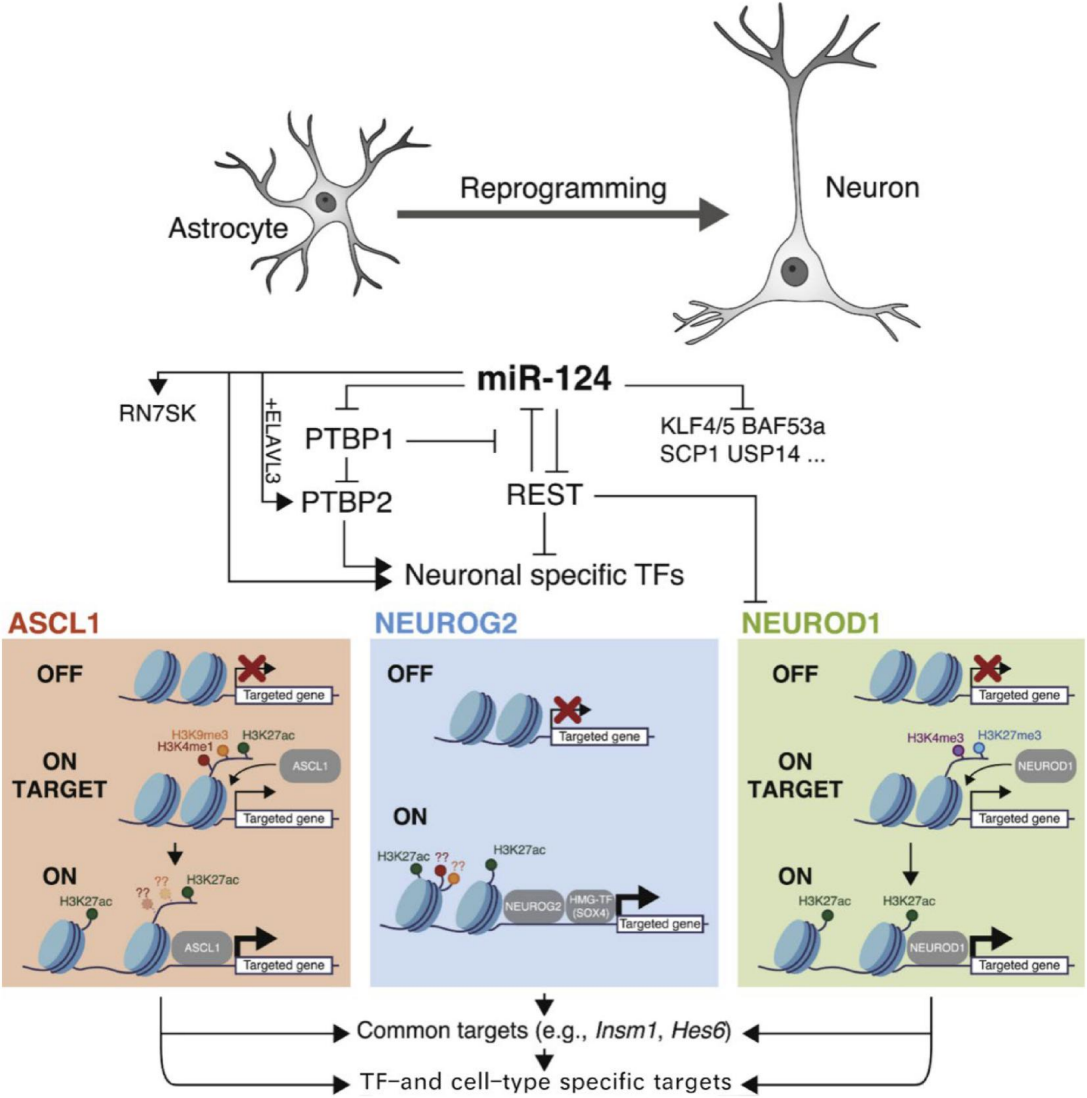
Molecular mechanisms of direct neuronal reprogramming. The figure shows neurogenic factors and fate suppressors that play a crucial role in neuronal reprogramming in vitro, defining the hierarchical trajectory of the feedback loop and converging on the regulation of pioneer factors (e.g., ASCL1, NEUROG2, and NEUROD1). Pioneer cells' unique trivalent chromatin marker (H3K4me1, H3K9me3, and H3K27ac) predicts that ASCL1 can bind to promoter regions and initiate chromatin remodeling/opening. Binding to ASCL1 enhances the acetylation of H3K27. A similar increase in H3K27 acetylation was observed following NEUROG2 and NEUROD1 overexpression. In the latter case, NEUROD1 also leads to loss of the repressive histone marker H3K27me3 at the target site.

Spinal cord injury or neurodegeneration is a very destructive neurological injury that severely impacts the life of the patient. The two major problems faced during the treatment of spinal cord injury are neuronal loss and regenerative glial scarring that inhibits neuronal regeneration. Previous stem cell transplantation studies have shown low cell survival rates and faced rejection and ethical issues.^47^ In vivo neuronal reprogramming is a recently developed technique for neural injury repair that has made a breakthrough in regenerative medicine.^48^ Traditional means of reprogramming glial cells with the help of viruses are associated with the risk of uncontrolled differentiation and tumorigenicity. At the same time, previous small‐molecule reprogramming often has the disadvantage of being multistep, complex, and lengthy, and thus its application can be limited. Fernandes et al.^49^ developed a new chemical‐based approach in which a chemical mixture formulated with RepSox, DAPT, Y26732, CHIR99021, ruxolitinib, and SAG was used on the mouse astrocyte cell line C8‐D1a to rapidly, simply, and efficiently induce the reprogramming of astrocytes to neurons within 4 days with 82 ± 6% conversion efficiency. These reprogrammed cells gained a glutamatergic phenotype for the next 11 days and the neuronal function of the induced cells was demonstrated by identifying the KCL‐induced calcium flux. Little research has shown that adult fibroblasts (aHDF) transform into pluripotent neural precursors having the ability to efficiently differentiate into cortical neurons. In a study published by Edwards et al.^50^ chemically modified mRNAs encoding the protoneuronal genes SOX2 and PAX6 were combined with small‐molecule supplements to improve the efficacy of reprogramming. This combined strategy significantly improved the efficiency of direct derivation, as evidenced by transcriptional phenotyping of spectrum‐specific progenitors and markers of cortical neuronal development, and following exposure to defined differentiation media, functional glutamatergic neurons are produced. This offers a previously highly actionable and productive approach^51^ (Figure 7). Reprogramming of the nervous system uses endogenous cells (such as scar fibroblasts or neurotoxic glial cells) to generate neural replacement cells, where glial cells are considered to be the cell type most readily converted into neurons that can be reprogrammed to target proliferation in vitro and in vivo.

## 11 CELLULAR REPROGRAMMING IN WOUND HEALING

One of the most readily available cells in the skin, dermal fibroblasts has received much interest in cell therapy. The introduction of new modalities to develop cell‐based therapies for treating skin diseases using the regenerative capacity of dermal fibroblasts is gaining the attention of scholars.^52^ iPSC‐based treatments can be targeted at different stages of wound healing and are shown to accelerate wound healing by augmenting angiogenesis, regeneration, cell migration tissue, and modulating inflammation.^53^ Shen et al.^54^ prepared wound models in nude mice to study the effect of bioengineered vascularized structures as a therapeutic modality for diabetic wound healing, and they used fibroblasts derived from healthy donors or type 1 diabetic patients, reprogrammed them into hiPSC, and engineered vascularized structures based on endothelial progenitor cells or early vascular cells differentiated from them vascularized designs based on their differentiation from endothelial progenitor cells or early vascular cells to treat wounds in nude mice. They discovered that all angiated structures accelerated wound closing and reperfusion, where endothelial progenitor cell structures had the earliest maximum closure rates, with healthy and diabetic hiPSC‐derived designs following closely behind. It was accompanied by rapid formation and regression of the sarcolemma in nude mice in all revascularized structure groups. Reprogramming fibroblasts into iPSCs by gene editing is a promising approach for treating skin diseases.^55^ iPSC has revolutionized the arena of wound repair and skin tissue engineering. Although progress has been made, the security and heterogeneity of iPSC lines remain major hurdles and extensive animal studies are lacking.

## 12 CELLULAR REPROGRAMMING IN HAIR FOLLICLE REPAIR

**FIGURE 8:**
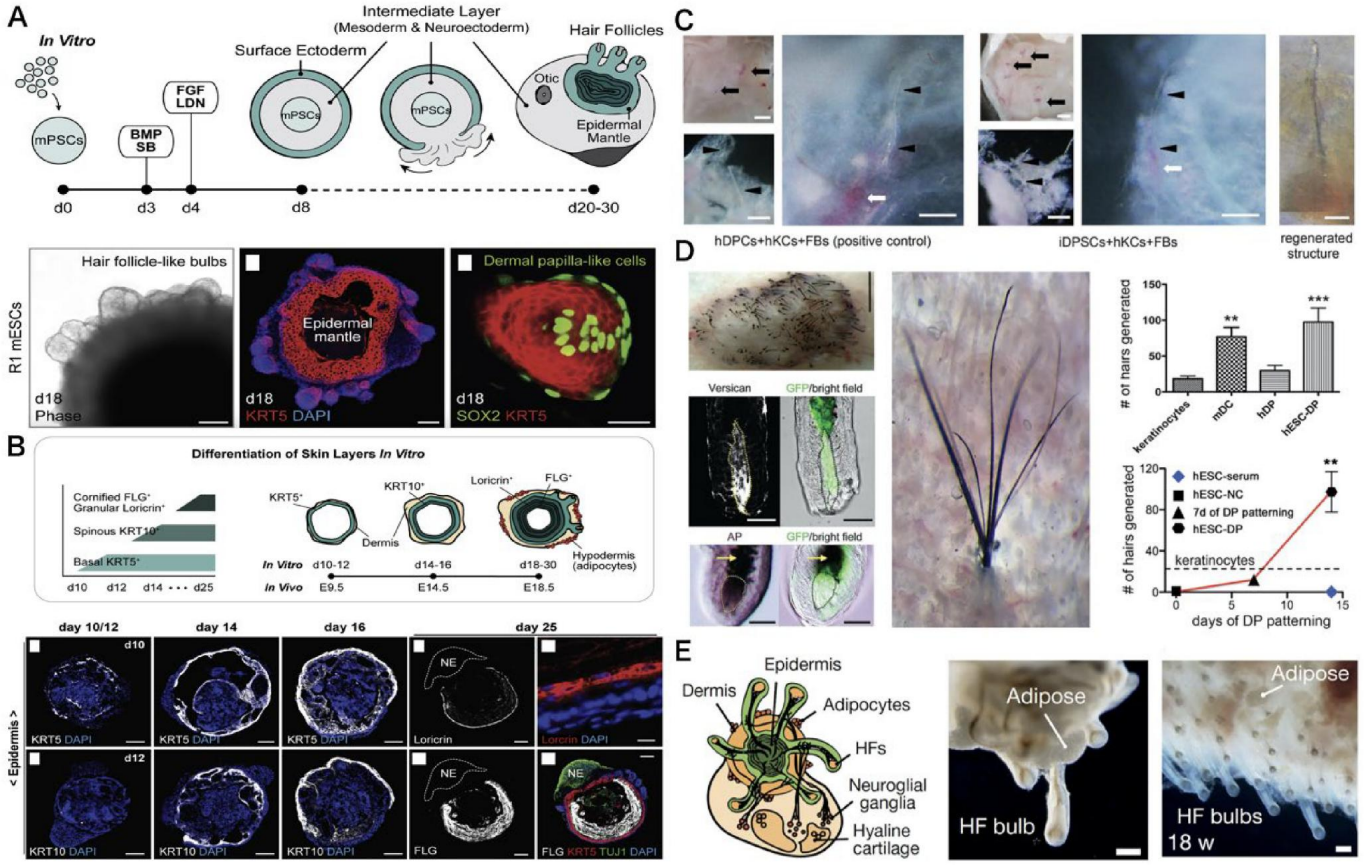
Hair follicle‐like organs with iDPSCs forming hair follicles. (A) Schematic overview of mouse embryonic stem cell culture of skin‐like organs. Representative phase‐contrast images of day 18 bulb‐like HFs are protruding from the surface of R1 mESC aggregates. Day 18 skin‐like organ cryosections showing representative IHC images of HF‐like bulbs of KRT5^+^ epithelial cells. (B) Schematic representation of skin developmental stages in vitro. Representative IHC images of self‐assembled epidermis on days 10–25. (C) *In vivo*, induced dermal papilla replacement cells (iDPSCs) exhibit functional DP properties. Co‐grafting of hKCs covered by FBs and hDPCs or iDPSCs produced cystic structures with focal aggregates (arrows) containing fine HF‐like structures (arrowheads), indicating DP properties of iDPSCs. Representative regenerated structures using the iDPSCs + hKC+ FB combination. hDPCs or iDPSCs were stained red with cytoplasmic orange membrane dye. (D) Folliculogenic capacity of human ES cell‐derived DP cells. Stereoscopic images of mouse keratin‐forming cells transplanted in combination with hESC‐DPC. Images of hair trunks from A after skin growth. GFP‐positive DPs of newly formed hairs are positive for Versican and alkaline phosphatase (AP). Quantification of keratin‐forming cell‐induced hairs transplanted alone or in combination with mDC, hDP, or hESC‐DP. Hair induction ability of ESC‐DP cells differentiated from hESC‐NC versus time. (E) Schematic representation of the structure of hair follicles in skin‐like organs induced to form by iDPSCs. Darkfield image of the skin‐like organ at day 140 showing fat and HF globules, human fetal forehead skin at 18 weeks, scale bar 250 μm.

Shinya Yamanaka's discovery of pluripotent stem cells in 2006 has made it possible to treat almost all human diseases with regenerative medicine, and hair loss may also see the light of day because of this therapy.^56^ Before applying iPSC for hair regrowth, Lee et al.^57^ reported that mouse embryonic PSCs will newborn hair follicles (HFs), outlining the critical steps in developing their derived skin‐like organs. Their results support a method of generating dermal and cutaneous appendages from PSCs in which epithelial and dermal cells are co‐triggered into an organoid unit (Figure 8A,B). Veraitch et al.^58^ overcame the technical limitations of the current preparation of human hair cells by using high proliferative and plastic human iPSCs (Figure 8C). A bone marrow stromal cell phenotype was obtained by differentiation of hiPSCs into induced mesenchymal stromal cells (iMCs). Using retinoic acid and DP cell activation medium, a hyperproliferative and plastic LNGFR(+)THY‐1(+) subpopulation of iMCs was programmed for DP properties. The induced DP replacement cells (iDPSCs) displayed upregulated DP markers and induced human keratin‐forming cells to express HF genes, which, when co‐transplanted in vivo with human keratin‐forming cells, produced fibrillar structures with a hair cuticle coating similar to that of the hair shaft as confirmed by the scanning electron microscopy analysis. Autologous iPSC generated after reprogramming somatic cells from alopecia areata patients on mouse embryonic stem cells can be expanded and cryopreserved.^59^ These iPSCs can then differentiate into a wide range of cell types, including melanocytes, epithelial cells, dermal papillae, and other cell types that make up functioning hair follicles. iPSC‐derived follicular cells transplanted into nude mice by Gnedeva et al.^60^ have successfully produced xenografts with hair growth (Figure 8D). Since iPSC provides a virtually limitless supply of follicle‐producing cells for hair follicle formation from the outset, this approach offers significant benefits compared to current surgical hair transplantation surgery, as the latter simply redistributes existing hair follicles from one site to another. In conjunction with robotics and the simulation of the grafting process, this new approach to rejuvenation medicine could well make hair restoration a routine operation that everyone can afford and benefit from Lee et al.^61^ (Figure 8E).

## 13 CELLULAR REPROGRAMMING IN INNER EAR REPAIR

**FIGURE 9:**
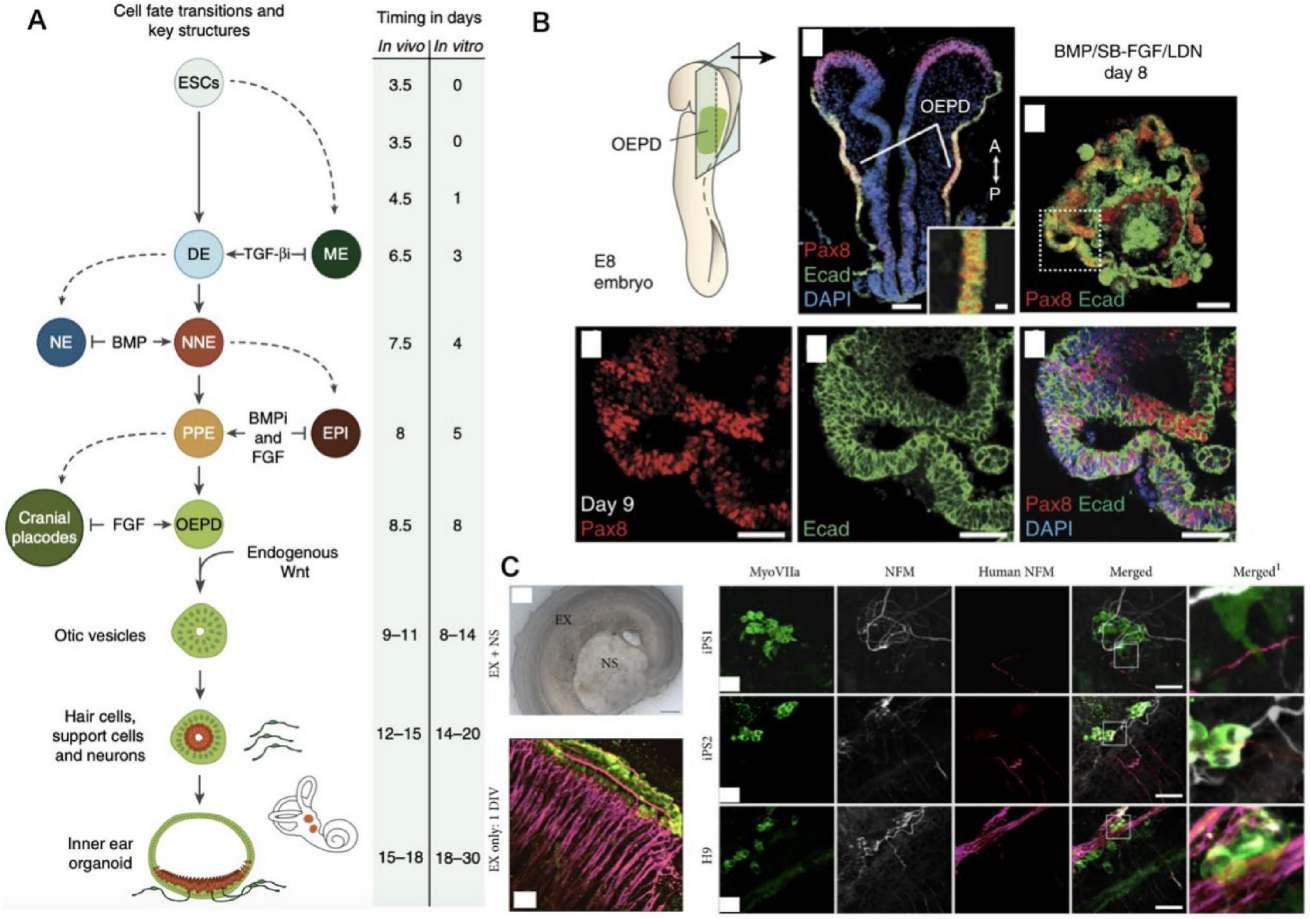
iPSCs in inner ear repair. (A) Comparison of cell fate and morphological transformations during inner ear development in vivo and in vitro. (B) Epithelium of OEPD and outer epithelium of BMP/SB‐FGF/LDN treated aggregates in E8 mouse embryos expressing Pax8 and Ecad. (C) Cochlear explants were co‐cultured with hESC and hiPSC‐derived neural precursor cells—photomicroscopic images showing cochlear explants co‐cultured with stem cell‐derived neurospheres (NS). Exosomal controls obtained from P3 mice offer normal innervation patterns of ANs after 1 day of in vitro culture. In co‐culture, hESC‐ and hiPSC‐derived neuro‐protuberances were observed to grow along with the hair cell arrangement. Myosin (VIIa; green) labels hair cells, neurofilaments (NFM; gray) label endogenous neural processes, and human NFM (hNFM; red) label stem cell‐derived neural processes. Merged1 images represent high magnification images of inserts depicting contacts between stem cell‐derived neural processes and hair cells.

It contains sensory epithelial cells that allow the detection of head movements, gravity, and sound. Damage to the inner ear can severely impair hearing and balance disorders. There is a lack of these studies using pluripotent stem cells to develop these cells. The failure to obtain sensory neurons and mechanosensitive hair cells in the inner ear often results in poor conversion efficiency or partial phenotypic conversion of derived stem cells. There was no understanding of the importance of the non‐neural and preplacental ectoderm, the two fundamental precursors of the inner ear, in previous studies.^62^ (Figure 9A). Koehler et al.^63^ reported the progressive derivation of mouse ESCs into inner ear sensory epithelial cells within a three‐dimensional medium. They show that ESC clusters are converted sequentially into non‐neural, pre‐stromal, and ear stromal epithelial cells through precise timing of signaling pathways to control development in vivo. Remarkably, vesicles containing pre‐sensory cells appear from the presumed ear stroma in a self‐organizing procedure mimicking normal development and generate hair cells with static and motor cilia bundles. They also display functional characteristics of natural mechanosensitive hair cells and form dedicated synapses with sensory neurons derived from ESCs, in architecture and biochemistry, the vesicles resemble the growing vestibular end organ. iPSCs, if successfully differentiated and reconnected to remnants of the damaged auditory system, could act as an autologous origin of damaged cochlear replacement neurons. Gunewardene et al.^64^ used established in vitro experiments to investigate the neural stimulation potential of hiPSC‐derived neurons on early postnatal hair cells (Figure 9B). They compared two sets of hiPSC with a better characterized set of hESC. Following 10 days of in vitro co‐culture, hiPSC‐derived neural processes throughout the cochlear explants were in contact with internal and external hair cells and showed expression of synaptic proteins in these contact points. Importantly, hiPSC‐derived neurons in early differentiation co‐cultured with hair cells formed more pronounced synapses with hair cells than did hiPSC‐derived neurons co‐cultured in late differentiation. hiPSC‐derived neurons also differed significantly in their innervation capacity as well as in innervation efficiency between lineages. Overall, such data demonstrate the potential of hiPSCs to replace auditory neurons and emphasize the importance of developing methods to mitigate the observed differences between hiPSC cell lineages to achieve dependable clinical improvement for patients. Cell replacement therapies using human iPSC or ESC hold great promise. Some of the limiting factors that hindered the progress of stem cell therapy, including low yield and heterogeneity of differentiated cells, are gradually being overcome. The efficient differentiation of many functional hair cells and spiral ganglion neurons (SGN) has been promoted, laying the foundation for iPSC to be widely used for inner ear repair.

## 14 CELLULAR REPROGRAMMING IN SKELETAL MUSCLE REPAIR

The susceptibility of skeletal muscle to mechanical stimulation is evidenced, for example, in the increase in muscle cell bulk due to hypertrophy of raw muscle fibers after regular weight lifting. On the other hand, prolonged immobility may eventually lead to atrophy of the muscles, one reason being the absence of physical stimulation. Therefore, physical stimulation is necessary for the maintenance of adult skeletal muscle. However, it is also the case that they are essential stimuli during muscle development and regeneration. Therefore, mechanical stimulation in vitro was already used as a bionic input to improve the maturation and shrinkage of engineered muscles.^65^ One of the core challenges of tissue engineering is to obtain appropriate cells and to use suitable biomaterials as tissue mimics to replace or repair damaged or diseased tissues. Remarkably, in the case of skeletal muscle tissue engineering, there is an urgent need to find safe sources of regenerative cells due to the lack of usability and regenerative capacity of endogenous myogenic cells and the tumorigenic risk they may pose. iPSCs could generate myogenic progenitor cells and thus overcome this problem. Rao et al.^66^ demonstrated the acquisition of iMPC by instantaneous over‐expression of Pax7 in proximal mesodermal cells differentiated from human pluripotent stem cells (hPSC), resulting in functional skeletal muscle tissue. In two‐dimensional culture, iMPC easily differentiates into spontaneously contracting multinucleated myotubes and a pool of satellite‐like cells that express Pax7 endogenously. However, under optimal 3D culture conditions, iMPC from multiple hPSC strains can reproducibly form professional skeletal muscle tissue consisting of well‐aligned multinucleated myotubes (iSKM bundles) showing positive force‐frequency relationships and strong calcium transients following electrical or acetylcholine stimulation. Throughout the 1‐month culture, iSKM exhibited structural hypertrophy, molecular maturation, and force production. When implanted in back or hind limb muscles of the mouse, iSKM could survive, gradually form blood vessels, and maintain the function. Yang et al.^67^ established a reprogramming technique for reprogramming dermal fibroblasts into pluripotent cells with a single protein, fibrillar regulatory protein. The resulting FMOD reprogrammed cells exhibit significant myogenic capacity with no risk of tumorigenicity, rendering them a hopeful source for cytoskeletal muscle reconstruction. For reconstructing muscle tissue and using optimal cells, maintaining cellular localization and retention in the receptor tissue environment is equally important. In the research, they showed that photopolymerizable methacrylate glycol chitosan (MeGC)/type I collagen (ColI)‐hydrogels provide an ideal encapsulated microenvironment for FReP cells, with survival, extension, spreading, and formation of myotubes in 3D hydrogels in vitro with no extra chondrogenic, osteogenic, or tendon differentiation. These findings prove that FReP cell‐MeGC/ColI hydrogel constructs are hopeful in vitro skeletal myogenic tissue engineering mimic. Conversion of the spectrum from one kind of somatic cell to another is an appealing way to obtain particular therapeutic cell generation. Transdifferentiation/direct transformation techniques have been recently developed to bypass the induced pluripotency stage. Hong et al.^68^ reported the invention of a method of direct transformation in which cells are transdifferentiated by exposure to an intermediate state of plasticity induced by a reprogramming factor based on nonintegrating small circular DNA (MCDNA) and then differentiated into adult myoblasts, a method that tends to have low delivery efficiency. To improve the delivery efficiency of MCDNA, they delivered reprogramming factors into chondrocytes by electroporation followed by transfection with poly(β‐amino ester) (PBAE). They used this method to transform human chondrocytes into myogenic cells and activate the vegetal receptor‐like kinase receptor inhibitors by treatment with SB‐431542 for enhancing myogenic. Differentiated cells exhibit myogenic marker expression, like Myod and Myog. This direct lineage transformation from chondrocytes to myogenic cells presents a new nonviral approach to transforming difficult‐to‐transfect cells into others of the lineage. iPSCs can generate myogenic progenitor cells, and combining them with bioengineering strategies to regulate their phenotype is understudied. The potential of such combinations is highlighted in this review, which aims to advance skeletal muscle tissue engineering.

## 15 CELLULAR REPROGRAMMING IN INTERVERTEBRAL DISC REPAIR

Degeneration of the nucleus pulposus (NP) is the first sign of intervertebral disc (IVD) degeneration, which gradually degenerates with age after birth, resulting in back pain.^69^ Cell‐based therapies could be generated by generating iPSCs that differentiate into nucleus pulposus‐like cells, according to recent studies on human iPSCs. Tang et al.^70^ used a lentivirus containing a transcription factor brachyury (T) promoter with a fluorescent reporter construct of green fluorescent protein (GFP) to transduce hiPSCs, followed by classification of T expression according to GFP intensity by flow cytometry. In vitro differentiation of hiPSCs into NP‐like cells was successful. Post‐induction pellet culture promoted vacuolation and surface marker expression in NP cells, LMα5, CD24, and Basp1 enrichment of brachyury (T)‐positive cells improved NP‐like cell differentiation efficiency further. Insight into NP cell differentiation might be gained by demonstrating that human iPSCs can be efficiently differentiated into NP‐like cells and potential sources of new IVD treatment cells. Seki et al.^71^ used normal human skin fibroblasts (NHDFs) to induce differentiation into bone marrow (NP) cells. They evidenced ectopic expression of MYC, KLF4, NOTO, SOX5, SOX6, and SOX9 in NHDFs using a three‐dimensional culture system to generate NP‐like cells, with Fandango‐O staining, qPCR, microarray analysis, and fluorescence‐activated cell sorting, it was determined that induced NP cells show a fully differentiated phenotype. IVD cell‐based therapy has become something of an exciting field, where studies report the regenerative possibilities of lots of cell sources, ranging from autologous or allogeneic chondrocytes to native IVD cells and stem cells. This class of drugs is capable of inducing efficient differentiation of pluripotent stem cells into NP‐like cells; as a result, new therapies for treating diseases associated with IVD may be found by exploring the differentiation process of NP cells. IVD diseases may be treated more efficiently by these technologies.

## 16 CELLULAR REPROGRAMMING IN BONE REPAIR

**FIGURE 10:**
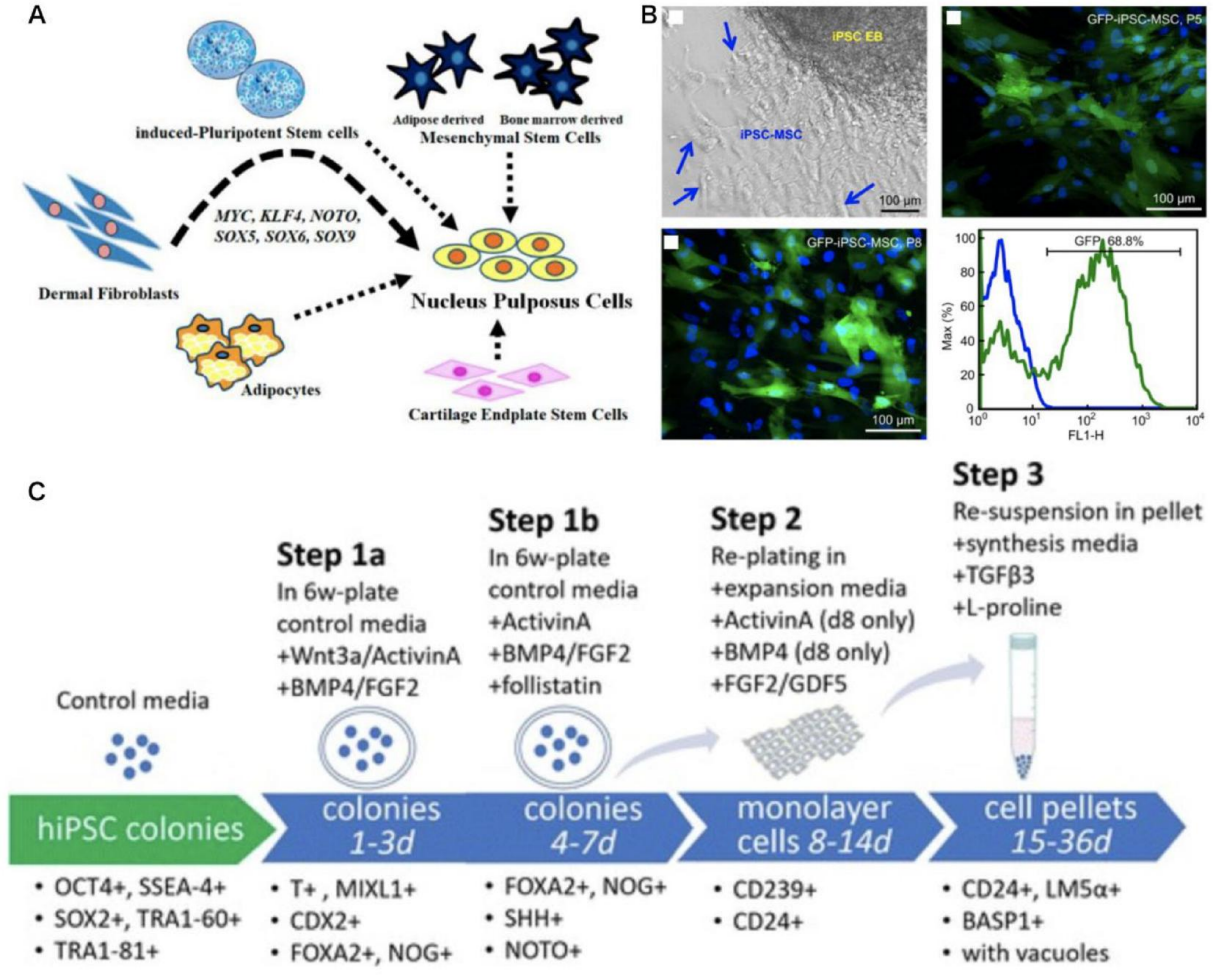
iPSC‐MSCs used with intervertebral disc repair. (A) Potential targets of myeloid origin. (B) Derivation and lentiviral transduction of iPSC‐MSCs. MSCs migrated from iPSC embryoid bodies (EBs). Arrows indicate iPSC‐MSCs, which were later harvested and expanded for experimental use. GFP‐iPSC‐MSCs were examined using fluorescence microscopy. GFP is green, and the nuclei are blue. Green fluorescence was constantly detected in fifth to eighth generation GFP‐iPSC‐MSCs. (C) Schematic representation of the three steps of directed differentiation of undifferentiated hiPSCs into np‐like cells.

A critical and challenging problem in clinical practice is bone regeneration. Bone tissue engineering (BTE) cell sources can offer an optimal tactic for reconstruction. Mao et al.^72^ investigated whether iPSC‐derived adipocytes (ASCs) will be used as osteogenic substitutes and if these ASC‐iPSCs would produce more formation of new bone than ASCs in hydrogel scaffolds (Figure 10A). Therefore, they reprogrammed ASCs into ASC‐iPSCs by a retroviral system and seeded ASCs and ASC‐iPSCs directly onto cryogel scaffolds of nHAP‐gelatin. Dorsal implantations of cell scaffolds in nude mice were used to compare the osteogenic potential between ASCs and ASC‐iPSCs in vivo. PCR results showed that ASC‐iPSCs expressed specific genes capable of causing differentiation into the three germ layers of iPSCs 4 and 8 weeks after implantation. The results suggest that ASC‐iPSCs may be excellent sources of BTE cells with better osteogenic differentiation efficacy for future medical applications. iMSCs‐derived pluripotent stem cells are another promising option for patient specificity. Bone iPSC‐MSCs modified with the BMP2 gene have not been reported yet for bone tissue engineering. Liu et al.^73^ genetically modified iPSC‐MSCs for BMP2 delivery by green fluorescent protein GFP‐iPSC‐MSCs or BMP2‐iPSC‐MSCs infection and inoculated BMP2 gene‐modified iPSC‐MSCs bone tissue engineering on CPC fixed with RGD RT‐PCR, and ELISA results showed that BMP2‐iPSC‐MSCs showed higher levels of BMP2 than iPSC‐MSCs (*p* < 0.05) and did not impair cell growth compared to iPSC‐MSC (Figure 10B). After 14 days of culture in an osteogenic medium, the ALP activity of BMP2‐iPSC‐MSCs was 1.8‐fold higher than that of iPSC‐MSCs (*p* < 0.05), indicating that BMP2 gene transduction in iPSC‐MSCs improved osteogenic differentiation, COL1A1 expression was 1.9‐fold higher than that of iPSC‐MSCs, and OC expression was 2.3‐fold higher than that of iPSC‐MSCs. At 21 days, matrix mineralization of bone BMP2‐iPSC‐MSCs was 1.8 times higher than which of iPSC‐MSCs. In the conclusion, iPSC‐MSCs inoculated on CPC are appropriate for BTE. iPSC‐MSCs modified with BMP2 gene on RGD‐CPC enhanced bone differentiation and mineralization. Inoculation of BMP2‐iPSC‐MSCs on RGD‐CPC stents is expected to improve bone regeneration efficiency. Induced pluripotent stem (iPS) cells are regarded as an appealing option for osteogenic differentiation and bone regeneration. Indeed, it has been demonstrated that recent discoveries found that the existence of multiple ways iPS cells can differentiate into osteoblasts, like proteins, growth factors, cytokines, and small molecules, which suggests that iPS cells have the potential to drive future bone regeneration therapies.

## 17 CELLULAR REPROGRAMMING IN LIVER REPAIR

Hepatocyte‐like cells are essential for the treatment of terminal liver disease and nowadays may be obtained by a variety of reprogramming techniques. Nevertheless, it is a complex procedure to induce hepatocytes from iPSCs, and improving the differentiation state and physiological function of these cells remains challenging.^74^ The overexpression of cell lineage‐specific transcription factors can directly transform terminally differentiated cells into some other cell lineage, involving blood progenitors, cardiomyocytes, and neurons. Huang et al.^75^ induced functional iHep cells directly from mouse tail‐tip fibroblasts by transducing Foxa3, Hnf1α, and Gata4 and inactivating p19(Arf). iHep cells exhibited classic epithelial cell‐shaped expressed hepatic genes and acquired hepatocyte functions. Remarkably, transplanted iHep cells repopulated the livers of mice lacking fumarate acetylhydrolase (Fah (−/−)) and saved nearly half of the recipients from death by restoring liver function. This research offers a novel strategy to produce functional hepatocyte‐like cells for liver engineering and regenerative medicine. These genealogically transformed cells were shown to repair damaged liver tissue in vivo. Hishida et al.^76^ partially reprogrammed adult hepatocytes to the precursor cell state by inducing the specific expression of four Yamanaka factors in hepatocytes and the short‐term expression of 4F enhanced liver regeneration by topoisomerase 2‐mediated partial reprogramming. Reduced manifestation of differentiated liver lineage markers, increased expression of proliferation and chromatin modifier markers, overall alterations in DNA, and access to hepatic stem and progenitor cell markers were obtained. Such results suggest that in vivo expression of liver‐specific 4F induces cell plasticity and counteracts hepatic failure, indicating that partly reprogramming might be a way to promote tissue regeneration.

## 18 CELLULAR REPROGRAMMING IN KIDNEY REPAIR

A regenerative medicine strategy using renal progenitor cells (NPCs) is a feasible approach.^77^ Nevertheless, the production of induced renal progenitor cells (iNPCs) from human cells remains a significant difficulty. Mulder et al.^78^ isolated urine cells (UCs) from pediatric urine specimens and reprogrammed them into urine‐induced pluripotent stem cells (UiPSCs) using an episomal vector. After assessment of iPSC quality, human kidney‐like organs were produced. These UiPSCs succeeded in differentiating into kidney organs that closely resembled organs produced from iPSCs derived from control constitutive fibroblasts. It is an appealing method for patient‐specific iPSC studies concerning infants and children, having broad applicability and low participation thresholds. Gao et al.^79^ proposed a new approach to generate NPCs from UCs, that is, iNPCs were generated from 10 ml of human UCs by forcing the expression of transcription factors 4F and SLUG and then exposing them to several specific small molecules. The obtained iNPCs are similar to hESC‐derived NPCs concerning morphology, biological properties, the potential for differentiation, as well as overall gene expression, and these cells can undergo long‐term expansion under serum‐free conditions, which will contribute to their rapid, efficient, and broad applicability with patient specificity, especially with the potential to serve as a source of transplantable cells for patients with kidney disease.

## 19 SUMMARY AND OUTLOOK

The review provides an overview of recent research progress in iPSC induction methods and clinical applications. Starting with introducing the comparison between advanced reprogramming techniques and traditional reprogramming methods in the preparation of iPSCs, the mechanisms of generation of different iPSCs are introduced. Comprehensive approaches involving biological, chemical, and physical methods have been chosen for cell reprogramming. Numerous other methods have been employed to produce iPSC, like nuclear transplantation, mandatory expression of specific factors, and combinations of a few small molecule compounds. A variety of approaches have been discussed to bring reprogramming factors into cells to generate iPSC, which are broadly categorized as (i) integration approaches, where genes for transcription factors are inserted into the genome of the cells to be reprogrammed, and (ii) nonintegration approaches, where the cell genome is not disrupted. However, the efficiency of all these reprogramming methods is still as low as 1%–5%. Integration methods are the most commonly used techniques for iPSC generation because of their guarantee of continuous ectopic expression of reprogramming factors in the host cell. The delivery system involves the integration of reprogramming factors into the genome of the host cell using viral and nonviral vectors. Although the integration of reprogramming factors into the genome of the host cell has been demonstrated, the main drawback of this reprogramming technique is that it leads to insertional mutagenesis, which is undesirable. The novel induction of cellular reprogramming by physical and chemical techniques that are currently emerging may become a reality in the future.

For many disorders, that is, spinal cord injury, retinal injury, myocardial infarction, etc., although many different cell types have been investigated to promote injury repair and recovery, it seems that stem cells are the most hopeful. Somatic cells may be reprogrammed into iPSCs, which have potential applications in regenerative medicine. Here, we review experimental approaches to treat clinical diseases with pluripotent stem cells, a cell type that represents an attractive source for developing new cellular therapies due to its inherent plasticity, self‐renewal, and differentiation potential. However, the purity, maturity, homogeneity, and efficacy of iPSC are yet to be further evaluated. In the future, if the high‐quality patient‐derived iPSC can be produced rapidly, clinical applications of autologous transplantation are expected to be realized, although further research is needed and challenges to clinical applications of iPSC need to be overcome:

(1) Inefficient production: Following the initial pioneering work on iPSC production by retroviral transduction of 4F, several alternative approaches to obtaining iPSC have already been developed to improve the yield and safety of the process. Many other methods have been employed to generate iPSC, like nuclear transplantation, forced expression of specific factors, and combinations of a few small‐molecule compounds. However, the efficiency of all these reprogramming methods still needs to be improved.
(2) Heterogeneity: iPSCs can exhibit heterogeneity in their pluripotency, self‐renewal capacity, and other characteristics. Even when PSC cell lines are derived from a single cell, heterogeneity between cells within the cell line may emerge during long‐term culture. Furthermore, the expression levels of genes and proteins in PSC fluctuate continuously at a frequency of hours to days, and this heterogeneity reduces the reproducibility of studies. Therefore, there is a need to detect, mitigate, and control heterogeneity in experiments involving human PSC in future studies.
(3) Tumorigenicity: one of the most critical clinical barriers to iPSC is that the genes responsible for the maintenance and pluripotency induction are also associated with cancer progression and therefore have the risk of inducing tumor formation after transplantation.^80^ The oncogenic transformation of iPSC may also be due to the insertion of reprogramming vectors to overexpress pluripotency factors. Currently developed with no virus and no integration gene delivery methods, like adenovirus, free plasmids, piggyBac transposons, and various novel chemical or physical means can help reduce the risk of tumorigenesis.
(4) Administrative obstacles: Every country maintains its own administrative and ethical guidelines related to regenerative medicine. iPSC generation involves a large part of the development process for which there is no global consensus. It is essential to establish clear guidelines to monitor the safety and effectiveness of the manufacturing process. Equally essential is ensuring informed consent and privacy protection for donors who collect cells and tissues for iPSC generation and research.

## AUTHOR CONTRIBUTIONS

Jiayi Mao: preparation, creation, and presentation of the published work, specifically writing the initial draft. Qimanguli Saiding: management and coordination responsibility for the research activity planning and execution and presentation of the published work. Shutong Qian: presentation of the published work. Zhimo Liu: helped perform the analysis with constructive discussions. Binfan Zhao: presentation of the published work. Qiuyu Zhao: writing the initial draft. Bolun Lu: contributed to the conception of the study. Xiyuan Mao: presentation of the published work. Liucheng Zhang: contributed significantly to analysis and manuscript preparation. Yuguang Zhang: acquisition of the financial support for the project leading to this publication. Xiaoming Sun: development or design of methodology. Wenguo Cui: formulation or evolution of overarching research goals and aims.

## CONFLICT OF INTEREST

The authors declare no conflict of interest. Wenguo Cui is a member of the *Smart Medicine* editorial board.
